# Magnetically recoverable solid acid photocatalyst activated by white LED for sustainable high-yield synthesis of 5-ethoxymethylfurfural from biomass

**DOI:** 10.1038/s41598-025-27726-y

**Published:** 2025-11-27

**Authors:** Pouya Ghamari Kargar, Mehdi Hosseini

**Affiliations:** 1 Department of Chemistry, Faculty of Basic Sciences, Ayatollah Boroujerdi University, Boroujerd, Iran; 2 Biosensor and Energy Research Center, Ayatollah Boroujerdi University, Boroujerd, Iran

**Keywords:** 5-ethoxymethylfurfural, Etherification, Acid catalysis, TiO_2_, white LED, Chemistry, Energy science and technology, Engineering, Environmental sciences, Materials science, Nanoscience and technology

## Abstract

The transition toward renewable energy demands innovative pathways for upgrading biomass into high-value biofuels with minimal environmental impact. Among various biofuel candidates, 5-ethoxymethylfurfural (EMF) has emerged as a superior green fuel additive due to its high energy density, low volatility, and compatibility with existing fuel infrastructure. However, achieving high yields from renewable carbohydrate sources remains challenging, particularly under environmentally benign conditions. Herein, we present a magnetically recoverable solid acid photocatalyst, Fe_3_O_4_@TiO_2_-diamine-SO_3_H, engineered through TiO_2_ coating of Fe_3_O_4_ nanoparticles followed by covalent immobilization of 1,4-butanesultone via 3,5-diaminobenzoic acid. This tailored nanoarchitecture synergistically integrates firm Brønsted acidity, visible-light photocatalytic activity, and magnetic separability, enabling efficient etherification of HMF with ethanol and direct one-pot dehydration etherification of sugars under white LED illumination. The optimized process (100 °C, 1.5 h) delivered remarkable EMF yields of 97% from 5-hydroxymethylfurfural (HMF), 79% from fructose, and 65% from inulin in ethanolic solvent without any additional co-solvent. This approach offers several key advantages: (i) operational simplicity and minimal synthetic steps, (ii) use of abundant, inexpensive raw materials, (iii) mild, energy-efficient reaction conditions, and (iv) excellent catalyst reusability over six cycles with negligible activity loss. By uniting green solvent systems, visible-light activation, and magnetically assisted recovery, this work provides a scalable and sustainable platform for biomass-to-biofuel conversion. The exceptional performance and recyclability of Fe_3_O_4_@TiO_2_-diamine-SO_3_H underscore its strong potential for industrial application in next-generation renewable fuel production.

**Supplementary Information:**

The online version contains supplementary material available at 10.1038/s41598-025-27726-y.

## Introduction

**Scheme 1:**
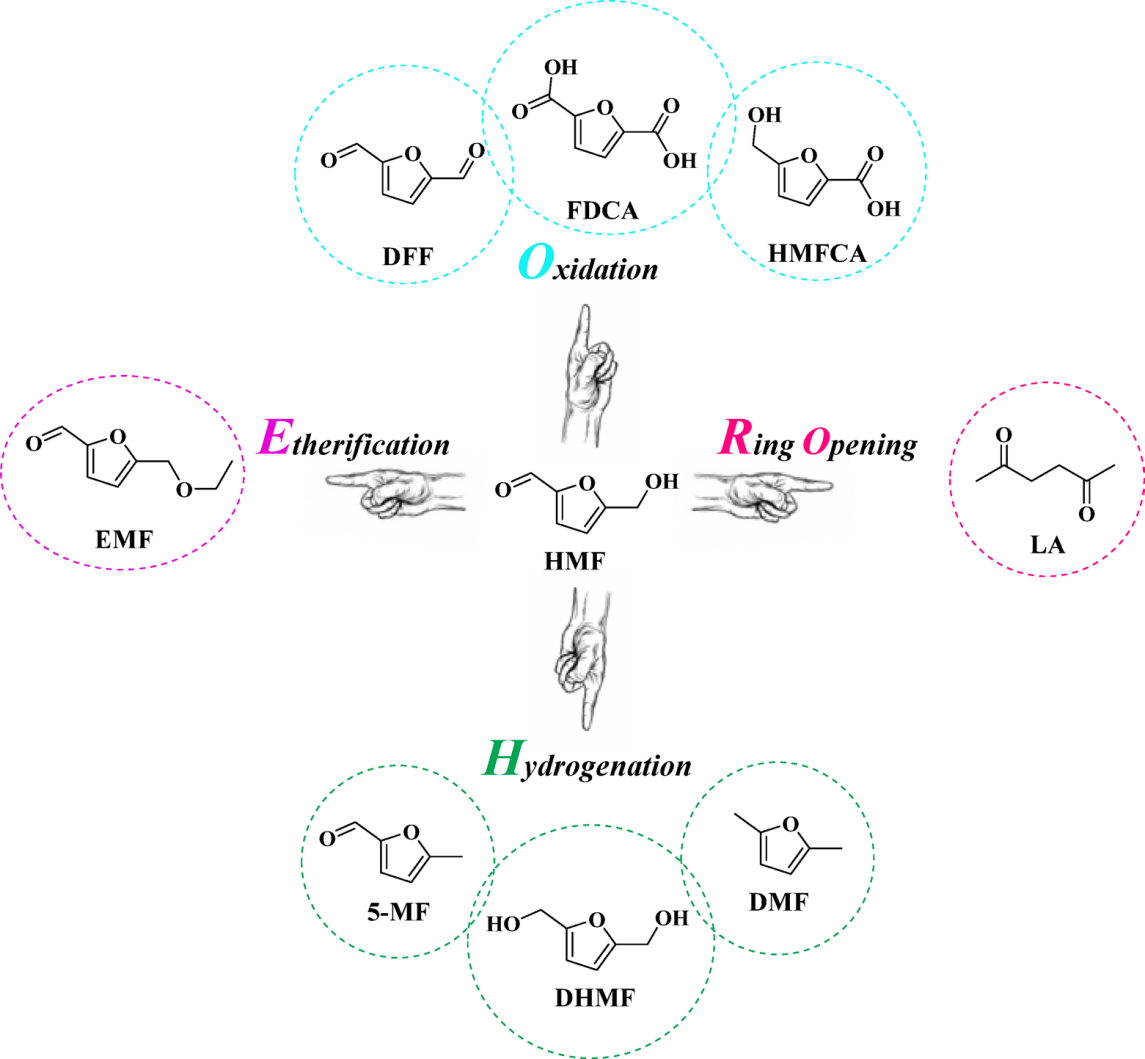
Showcases the spectrum of valuable chemicals accessible from HMF via catalytic transformations such as oxidation, ring opening, hydrogenation, and etherification^17–19^.

 Petroleum, coal, and natural gas remain the primary nonrenewable fossil fuels supporting industrial and societal energy needs^1–4^.Their extensive exploitation has led to energy security concerns and environmental issues, including pollution and climate change^5–7^. Therefore, transitioning to sustainable, low-carbon, renewable energy sources is a pressing scientific and technological challenge^8,9^. Biomass, due to its renewability, carbon neutrality, and wide availability, serves as a key feedstock for producing environmentally friendly chemicals and alternative fuels^10–12^. Heterogeneous catalysis offers advantages such as stability, recoverability, and scalability in these processes^13^. Among biomass-derived carbohydrates, saccharides like fructose, glucose, sucrose, inulin, and cellulose are important precursors for 5-hydroxymethylfurfural (HMF), a valuable platform molecule in biorefineries^14–17^. HMF, together with furfural, is recognized by the U.S. Department of Energy as a critical bio-based chemical building block for sustainable chemical production^18,19^. HMF is a versatile intermediate that can be converted into a wide range of high-value chemicals, enabling the production of renewable fuels and biodegradable materials^20–23^. Through various catalytic processes, HMF can be transformed into compounds such as FDCA, HMFCA, DFF, EMF, LA, DMF, DHMF, and 5-MF, as shown in Scheme 1^24–26^. Among these derivatives, furanic ethers like EMF, EFE, and BEMF have gained attention due to their high energy density, favorable cetane index, and compatibility with conventional fuels^27,28^. In particular, catalytic etherification of HMF to EMF is a promising route for producing next-generation biodiesel candidates that can enhance or partially replace petroleum-derived fuels^29–31^. Owing to its high energy density (8.7 kWh/L) on par with gasoline (8.8 kWh/L) and diesel (9.7 kWh/L) and far surpassing ethanol (6.1 kWh/L) EMF has garnered significant interest as a promising alternative fuel. Also, the high oxidation stability of EMF can effectively reduce the emissions of soot, NO_x_, and SO_x_^32–35^.

As a result, the synthesis of EMF has garnered considerable attention from both academic researchers and industry professionals^36,37^. The synthesis of EMF can be accomplished efficiently through the acid-catalyzed etherification of HMF, attaining high conversion and selectivity^38,39^. This transformation can also be executed within an integrated one-pot catalytic process, wherein readily available and low-cost carbohydrates serve as the initial feedstock, with HMF generated in situ as a key intermediate^40^. Such an approach offers distinct advantages, including superior atom economy, process intensification, and enhanced scalability for industrial deployment^41,42^. The catalytic conversion of biomass-derived carbohydrates to EMF generally proceeds through a sequential reaction network: (i) isomerization of glucose to fructose, (ii) dehydration of fructose to HMF, and (iii) etherification of HMF to EMF^43,44^. Over the past decade, significant progress has been made in optimizing these reactions using various catalytic systems. Reported catalysts include homogeneous, heterogeneous, and hybrid acid systems such as metal salts, sulfonated solid acids, functionalized ionic liquids, heteropoly acids, molecular sieves, and porous supports like zeolites, sulfonic acid-functionalized silica or carbon, and ion-exchange resins such as Amberlyst^13^. While these catalysts differ in activity, stability, and recyclability, they all aim to promote selective EMF production under green chemistry principles^45,46^. The introduction of sulfonic acid groups onto catalyst supports is a common strategy to enhance catalytic acidity and performance. This modification stabilizes active sites and allows tuning of acid site density by adjusting the type and amount of sulfonating agents^47,48^. Incorporating magnetic carriers into these frameworks enables easy catalyst separation using an external magnet and allows repeated reuse without significant activity loss^49,50^. Such magnetically recoverable solid acids provide operational simplicity and sustainability, making them highly suitable for biomass conversion and other green catalytic processes^51–53^.

Li et al. demonstrated that a family of SO₃H-functionalized polymeric catalysts could effectively promote the etherification of fructose to EMF^54^, achieving yields as high as 72.8% under reaction conditions of 110 °C for 10 h. In a related study, Ziliang et al. synthesized magnetically retrievable Fe_3_O_4_@C–SO_3_H catalysts and evaluated their performance in converting a range of carbohydrate substrates namely HMF, fructose, inulin, and sucrose into EMF, delivering yields of 88.4%, 67.8%, 58.4%, and 33.2%, respectively^55^. The notably lower efficiency observed with sucrose was attributed to its disaccharide composition of fructose and glucose, wherein fructose undergoes preferential conversion while glucose remains largely unreactive toward EMF formation. Shanshan et al. reported the fabrication of Fe_3_O_4_@SiO_2_-SH-Im-HSO_4_ catalysts via radical oligomerization of bis-vinyl imidazolium salts on mercaptopropyl-modified silica-coated Fe_3_O_4_ nanoparticles^56^. This multifunctional catalytic system exhibited an impressive EMF yield of 89.6% from HMF at 100 °C, and also facilitated single-step EMF production from fructose, sucrose, and inulin, yielding 60.4%, 34.4%, and 56.1%, respectively. Wang et al. developed sulfonic acid-functionalized ordered mesoporous carbon (OMC–SO_3_H) with high surface area and firm acidity for the conversion of fructose to either HMF or EMF^57^. Using DMSO as the reaction medium, the etherification of fructose to EMF proceeded at 120 °C within 30 min, yielding 89.4% HMF. In a prolonged single-step reaction (24 h) at 140 °C, EMF yields of 55.7%, 53.6%, and 26.8% were obtained from fructose, inulin, and sucrose, respectively. Additionally, a biomass-derived magnetic Fe_3_O_4_@C–SO_3_H catalyst, synthesized from wheat straw and incorporating –COOH, –SO_3_H, and –OH functionalities, delivered an EMF yield of 64.2% under standard conditions, which increased to 85.6% when employing a DMSO–EtOH binary solvent system for fructose etherification^58^.

Dai et al. reported a sulfonated nitrogen-containing polymer (SPC) catalyst, where dihydroxyacetone (DHA) disrupted existing hydrogen bonds between –SO₃H groups and polymer nitrogen atoms, forming new double hydrogen bonds with aromatic sulfonic groups and enabling fructose etherification to EMF with 68.8% yield^59^. Similarly, a porous sulfonated polystyrene-divinylbenzene material (PSDVB–SO₃H) achieved 67.5% EMF yield from fructose at 120 °C in 2 h, surpassing commercial Amberlyst-15, while methoxymethylfurfural (MMF), alkyl levulinates, and HMF were co-products (0–67% yield range). A two-step, one-pot strategy combining zeolite and PSDVB–SO₃H afforded EMF from glucose at 39.4% yield. FeVO₄-supported polyaniline sulfonated with chlorosulfonic acid yielded a multifunctional –SO₃H composite, efficiently converting sucrose, fructose, and HMF to EMF^60^. Gupta et al. developed a cost-effective carbocatalyst integrating Brønsted -SO_3_H and Lewis acidic Ti⁴⁺ sites, achieving 91% and 64% EMF yields from HMF and fructose, respectively, with ethyl levulinate as a byproduct^61^. Yan et al. prepared annealed bio-based carbon microspheres coated with PTFE (A-BCMSs–SO₃H@PTFE), producing over 60% EMF from HMF after 72 h^62^. Chen et al. introduced a bifunctional catalyst via Pickering HIPE templating, achieving 48.1% EMF from glucose in EtOH/THF^40^. Additionally, Hafizi et al. reported mesoporous SO₄²⁻/Al–Zr/KIT-6 composites with tunable ZrO₂/Al₂O₃ ratios and sulfation, showing 89.8% EMF yield with 99% HMF conversion^63^.

Building on our recent progress in magnetically recoverable photocatalysts, this study focuses on developing efficient systems for converting carbohydrates into 5-EMF^64–68^. By integrating high surface area, tunable acid–base sites, and magnetic recoverability, catalysts can achieve high efficiency, selectivity, and easy recyclability. Here, we designed a novel photocatalyst by covalently attaching 1,4-butanesultone onto TiO_2_-coated Fe_3_O_4_ nanoparticles using 3,5-diaminobenzoic acid as a linker, enhancing stability and functionality. The catalyst was evaluated for carbohydrate-to-EMF conversion, with key reaction parameters and reusability systematically studied. Its magnetic properties enabled simple separation and reuse without significant loss of activity, highlighting its potential for sustainable applications.

## Experimental

### Materials and instruments

All chemicals were obtained from Sigma suppliers and used without further purification. All the solvents were distilled and dried before use. The progress of the reactions and purity of the products were analyzed by TLC on silica gel PolyGram SILG/UV254 plates. Elemental analyses of C, H, N, and O were performed using a PerkinElmer CHNO elemental analyzer. A FTIR Tensor II was used to record Fourier transform infrared (FT-IR) plots of all the developed materials in the range of 4000–400 cm^−1^ using KBr. Transmission electron microscopy TEM was used to characterize the powder (Philips EM 208 S). The surface morphology, particle size, and shape of the catalyst were analyzed by field emission scanning electron microscopy (FE-SEM, Tescan MIRA4). The chemical structural integrity of the obtained nano catalyst was affirmed by energy-dispersive X-ray spectroscopy (EDX). Thermoanalyzer Shimadzu was used for thermogravimetric analysis (TGA) between 30 and 800 °C with a heating rate of 10 °C/min under a N_2_ flow. The X-ray diffraction (XRD) spectra were obtained by utilizing a Philips powder diffractometer type Rigaku Ultima IV, equipped with a graphite monochromator crystal. The X-ray wavelength deployed was 1.54 Å and the diffraction patterns were secured in the 2 h intervals (10–80) using a scanning rate of 1°/min. Magnetic measurements were carried out at ambient temperature using a vibrating sample magnetometer (VSM, LBKFB model). The surface area of the samples was analyzed by using a Micromeritics Tristar II 3020 based on Brunauer-Emmett- Diffuse reflectance UV-Vis spectra were taken using a UV-Vis spectrophotometer (UV-VIS CARRY100). The quantification of 5-(ethoxymethyl)furfural (5-EMF) and 5-hydroxymethylfurfural (5-HMF) in the reaction mixture was carried out using high-performance liquid chromatography (HPLC). Analyses were performed on a Shimadzu LC-20AD HPLC system equipped with a quaternary pump, an autosampler, a column oven, and a UV–Vis diode array detector (DAD). High-performance liquid chromatography (HPLC) was employed to identify and quantify the reaction products. The analyses were carried out on an Agilent 1200 series HPLC system equipped with a quaternary pump, an autosampler, a column oven, and a UV–Vis diode array detector (DAD). Chromatographic separation was achieved using a C18 reversed-phase column (Zorbax Eclipse XDB-C18, 4.6 × 250 mm, 5 μm particle size). The mobile phase consisted of water (solvent A) and acetonitrile (solvent B) in an isocratic ratio of 30:70 (v/v). The flow rate was maintained at 1.0 mL/min, and the injection volume was 20 µL. The column oven was kept at 30 °C, and detection was carried out at λ = 280 nm, corresponding to the maximum absorbance of both HMF and EMF. Data acquisition and processing were performed using Agilent ChemStation software. Under these optimized conditions, standard 5-HMF and 5-EMF exhibited retention times of 2.6 min and 3.0 min, respectively. The HPLC chromatogram of the synthesized sample revealed a dominant peak at 3.0 min, corresponding to EMF, with negligible residual HMF, confirming a 98% yield of EMF.

### Synthesis of Fe_3_O_4,_ TiO_2,_ Fe_3_O_4_@TiO_2_

Fe_3_O_4_ magnetic nanoparticles^69–71^, TiO_2_^72^, and Fe_3_O_4_ coated with TiO_2_ (Fe_3_O_4_@TiO_2_)^73^ were produced utilizing a method that has been detailed in prior literature.

### Synthesis of Fe_3_O_4_@TiO_2_-diamine

The Fe_3_O_4_@TiO_2_ nanoparticles (1.0 g) were dispersed ultrasonically in 100 ml of ethanol using ultrasonic irradiation for 30 min. Subsequently, a dropwise addition of an ethanolic solution containing 3,5-diaminobenzoic acid (2.5 mmol in 10 ml of ethanol) was made to the resulting suspension. After stirring the mixture for 6 h at reflux temperature, the Fe_3_O_4_@TiO_2_-diamine particles were magnetically separated from the suspension, followed by washing with deionized water and ethanol. The final magnetic nanoparticles were then dried in a vacuum oven at 50 °C for 6 h.

### Synthesis of Fe_3_O_4_@TiO_2_-diamine-SO_3_H

To synthesize the Fe_3_O_4_@TiO_2_-diamine-SO_3_H catalyst, Fe_3_O_4_@TiO_2_-diamine (1 g) is first dispersed in 30 mL of an aqueous solution containing 4 mmol of ClSO_3_H under ultrasonication for 30 min. The resultant suspension is then stirred at room temperature for 24 h. After this period, the mixture is treated with a carefully measured amount of chlorosulfonic acid (ClSO_3_H) to modify the amine groups and introduce sulfonic acid functionality. The Fe_3_O_4_@TiO_2_-diamine is added to the ClSO_3_H solution, and the reaction mixture is stirred to achieve homogeneity before heating it under mild conditions for 1 to 3 h, monitoring the progress of the reaction. Once the reaction is completed, the solid acid catalyst (Fe_3_O_4_@TiO_2_-diamine-SO_3_H) is isolated using a magnet, rinsed several times with water and acetonitrile to ensure the complete removal of byproducts like KCl, and finally dried under a vacuum oven at 50 °C for 6–8 h.

### Synthesis of EMF from Fructose and HMF over Fe_3_O_4_@TiO_2_-diamine-SO_3_H

The catalytic reactions were carried out in a cylindrical quartz batch reactor (inner volume 10 mL) equipped with a magnetic stirrer to ensure uniform mixing. A suspension containing fructose or HMF (1 mmol), ethanol (4 mL), and catalyst (10–40 mg) was introduced into the reactor. The system was simultaneously heated using an oil bath equipped with a digital thermostat to maintain the reaction temperature (100 °C). At the same time, the reactor was irradiated externally with a white LED lamp (5–20 W) placed at a fixed distance of 5 cm from the reactor wall. The reaction mixture was magnetically stirred during the process to guarantee homogeneous light exposure and temperature distribution. Control experiments were performed under dark conditions (heating only) and under light without heating to confirm the synergistic requirement of simultaneous irradiation and heating. After the reaction, the mixture was rapidly cooled to ambient temperature using an ice bath. The solid catalyst was recovered magnetically, and the resulting solution was subjected to quantitative analysis via HPLC (Fig. S1) and CHNO analysis. To ensure reproducibility, all reported data represent the average of at least two independent runs. The analysis and formulas employed for calculating substrate conversion, EMF yield, and EMF selectivity can be found in the Supplementary Material file.

### HPLC analysis

High-performance liquid chromatography (HPLC) analysis was performed to confirm the successful conversion of fructose-derived 5-hydroxymethylfurfural (HMF) into 5-ethoxymethylfurfural (EMF) (Fig. S1). Standard solutions of pure HMF and EMF were first analyzed to determine the retention times of each compound under the employed chromatographic conditions. The chromatogram of the HMF standard exhibited a sharp and well-resolved peak at 2.6 min (red trace), while the EMF standard showed a significant peak at 3.0 min (blue trace). Subsequently, the synthesized product mixture was subjected to HPLC analysis. As shown in the chromatogram (black trace), the spectrum is dominated by a single strong peak at 3.0 min, which corresponds to EMF, accompanied by a negligible signal at 2.6 min, indicating that almost all of the HMF precursor was consumed during the reaction. Notably, no additional peaks corresponding to side-products were detected, confirming the high selectivity of the catalytic system. Quantitative analysis based on peak area comparison revealed that the conversion of HMF to EMF reached 98% yield, which demonstrates the high efficiency of the developed catalytic process. These results unambiguously confirm that the designed catalyst selectively promoted the etherification of HMF, yielding EMF with excellent efficiency and purity.

## Result and discussion

### Synthesis and characterization of the solid acid catalyst

**Scheme 2:**
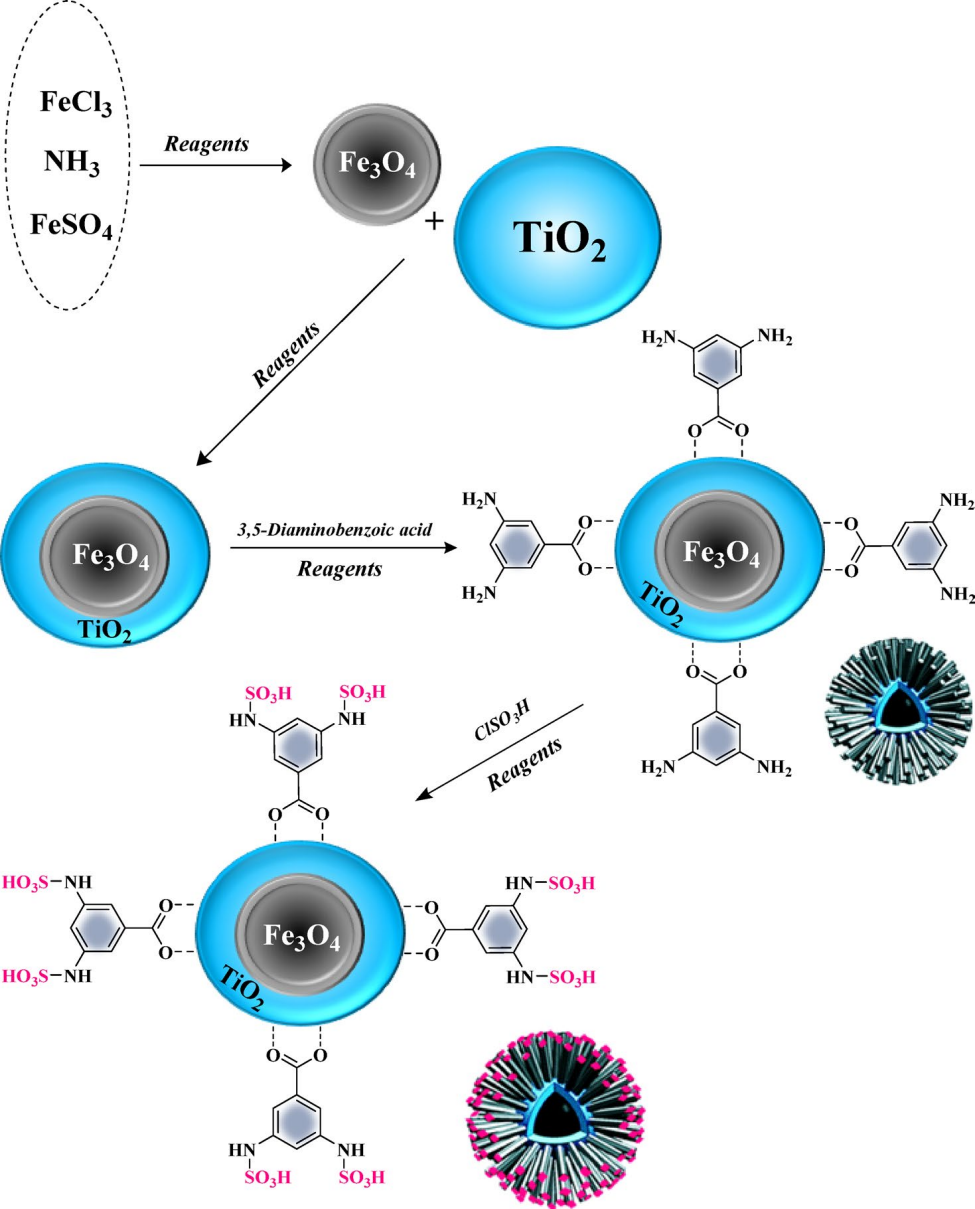
General route for preparation Fe_3_O_4_@TiO_2_-diamine-SO_3_H.

A multistep strategy was employed to prepare the solid acid catalyst, as outlined in Scheme 2. Initially, iron oxide nanoparticles (Fe_3_O_4_) were prepared as the catalyst backbone by mixing iron salts with a potassium solution and subjecting them to high-temperature calcination. Subsequently, a layer of titanium dioxide (TiO_2_) was applied on the iron oxide surface using a sol-gel method, enhancing the catalytic surface area and photocatalytic properties. In the next step, sulfonic acid functionalized diamine groups were introduced to the TiO_2_ surface through a chemical reaction, which improved the acidic and catalytic characteristics of the catalyst (Scheme 2). Finally, the resulting catalyst was subjected to magnetic separation techniques to ensure its proper functionality and stability during photocatalytic reactions. The final catalyst was then tested for its photocatalytic performance under white light irradiation. The acid loading content on Fe_3_O_4_@TiO_2_-diamine-SO_3_H was determined using acid-base titration, resulting in a value of 1.25 mmol/g. Additionally, elemental analysis confirmed a sulfur content of 4.20% in Fe_3_O_4_@TiO_2_-diamine-SO_3_H, corresponding to an acid loading of 1.30 mmol/g, which is consistent with the acid-base titration results (acidity measurement shown in supplementary material file).

**Fig. 1:**
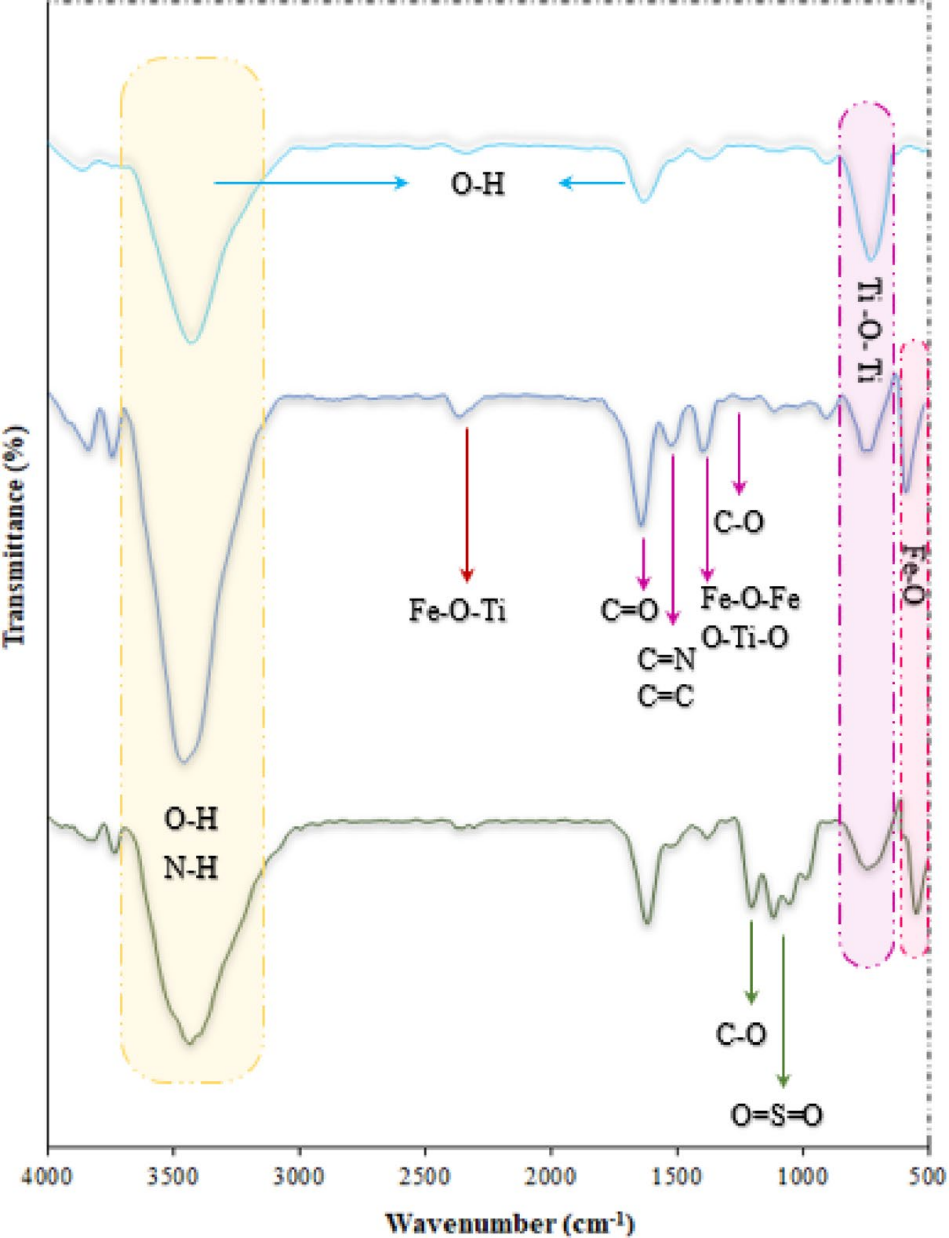
FT-IR spectra of TiO_2_ **(A)**, Fe_3_O_4_@TiO_2_-diamine **(B)**, and Fe_3_O_4_@TiO_2_-diamine-SO_3_H **(C)**.

The FT-IR spectra of TiO_2_ (A), Fe_3_O_4_@TiO_2_-diamine (B), and Fe_3_O_4_@TiO_2_-diamine-SO_3_H (C) are shown in Fig. 1. In the spectrum of pure TiO_2_ (Fig. 1A), a broad absorption band around 3100–3600 cm⁻¹ is attributed to the stretching vibrations of surface hydroxyl groups and adsorbed water molecules. In contrast, the characteristic Ti–O–Ti vibrations appear below 850 cm^−1^^74^. After Fe_3_O_4_ functionalization with TiO_2_, and diamine (Fig. 1B), additional absorption bands emerge: The N–H stretching mode overlaps with the O–H region (ca. 3200–3600 cm^−1^), and three distinct band at ~ 1200–1680 cm^−1^ is assigned to C-O, C = C, C = N, and C = O stretching, confirming the successful introduction of organic moieties. Furthermore, the band observed at 579 cm⁻¹ is attributed to the characteristic stretching of Fe–O bonds^75^. In the Fe-doped TiO_2_ structure, vibrations corresponding to O–Ti–O and symmetric Fe–O–Fe stretching appear at 1391 cm⁻¹^76^. Moreover, the peak at 2345 cm⁻¹ can be assigned to Ti–O–Fe linkages, indicating the chemical interaction established between the Fe_3_O_4_ magnetic core and the TiO_2_ shell during the doping process^77^. In the Fe_3_O_4_@TiO_2_-diamine-SO_3_H sample (Fig. 1C), the broad absorption observed near 3445 cm⁻¹ is indicative of O–H stretching vibrations from sulfonic acid groups. Two additional bands, appearing at 1140 and 1070 cm⁻¹, correspond to the stretching modes of SO₃^-^ and O = S = O, respectively. These features confirm the successful introduction of SO₃H functionalities onto the aromatic framework following the reaction of Fe_3_O_4_@TiO_2_-diamine with chlorosulfonic acid^78,79^. These observations collectively confirm the stepwise surface modification from TiO_2_ to Fe_3_O_4_@TiO_2_-diamine and finally to Fe_3_O_4_@TiO_2_-diamine-SO_3_H.

**Fig. 2:**
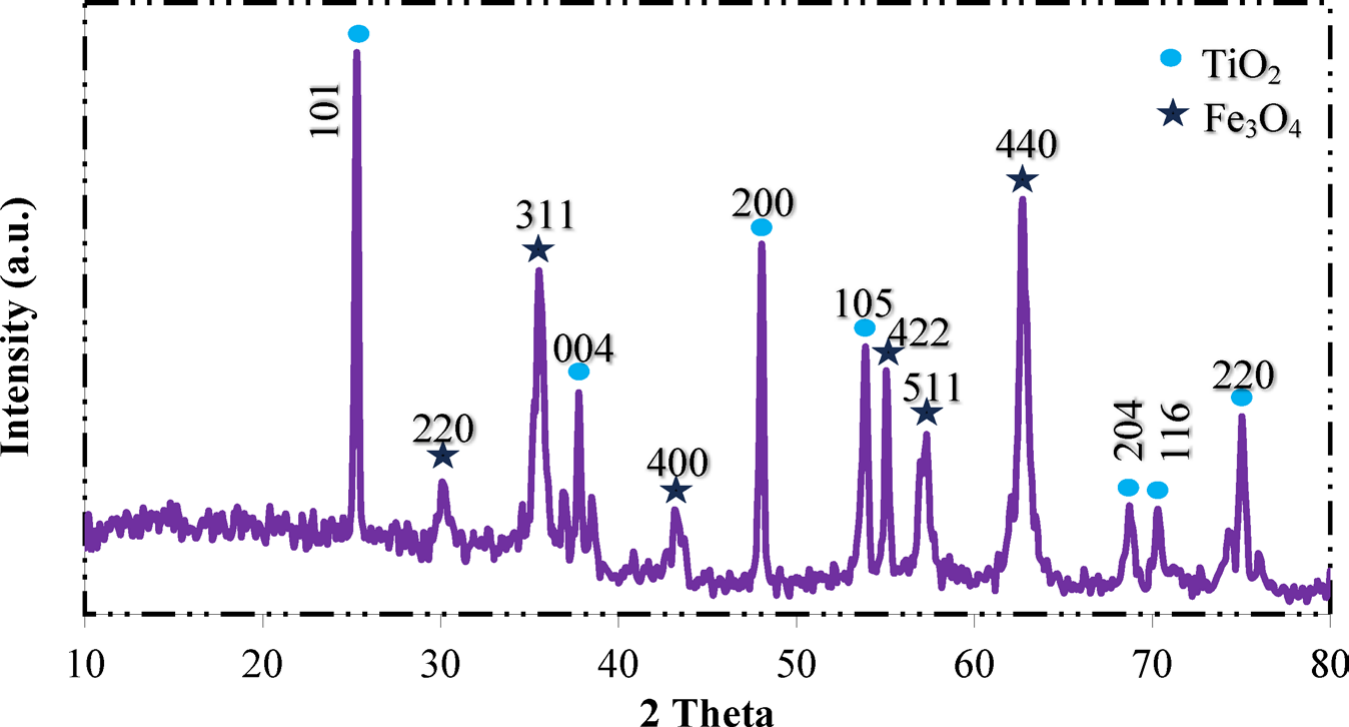
X-ray diffraction pattern of Fe_3_O_4_@TiO_2_-diamine-SO_3_H.

The XRD pattern of Fe_3_O_4_@TiO_2_-diamine-SO_3_H (Fig. 2) clearly displays the diffraction peaks corresponding to both TiO_2_ and Fe_3_O_4_ phases, confirming the successful fabrication of the magnetic core–shell composite. The intense reflections at 2θ = 25.3° (101), 37.8° (004), 48.0° (200), 54.0° (105), 55.1° (211), and 62.6° (204) can be indexed to the anatase phase of TiO_2_ (JCPDS No. 21–1272)^80^, while the peaks at 2θ = 30.1° (220), 35.6° (311), 43.3° (400), 55.0° (422), 57.0° (511) and 62.7° (440) are attributed to the inverse spinel structure of Fe_3_O_4_ (JCPDS No. 19–629)^81,82^. The coexistence of these two sets of reflections confirms that TiO_2_ has been successfully coated on the Fe_3_O_4_ surface without destroying the crystalline integrity of either component. Moreover, no additional impurity peaks were detected, suggesting that the functionalization with diamine and sulfonic acid groups did not alter the crystalline structures of Fe_3_O_4_ or TiO_2_. The preservation of crystallinity is expected to enhance the stability and catalytic activity of the composite material.

**Fig. 3:**
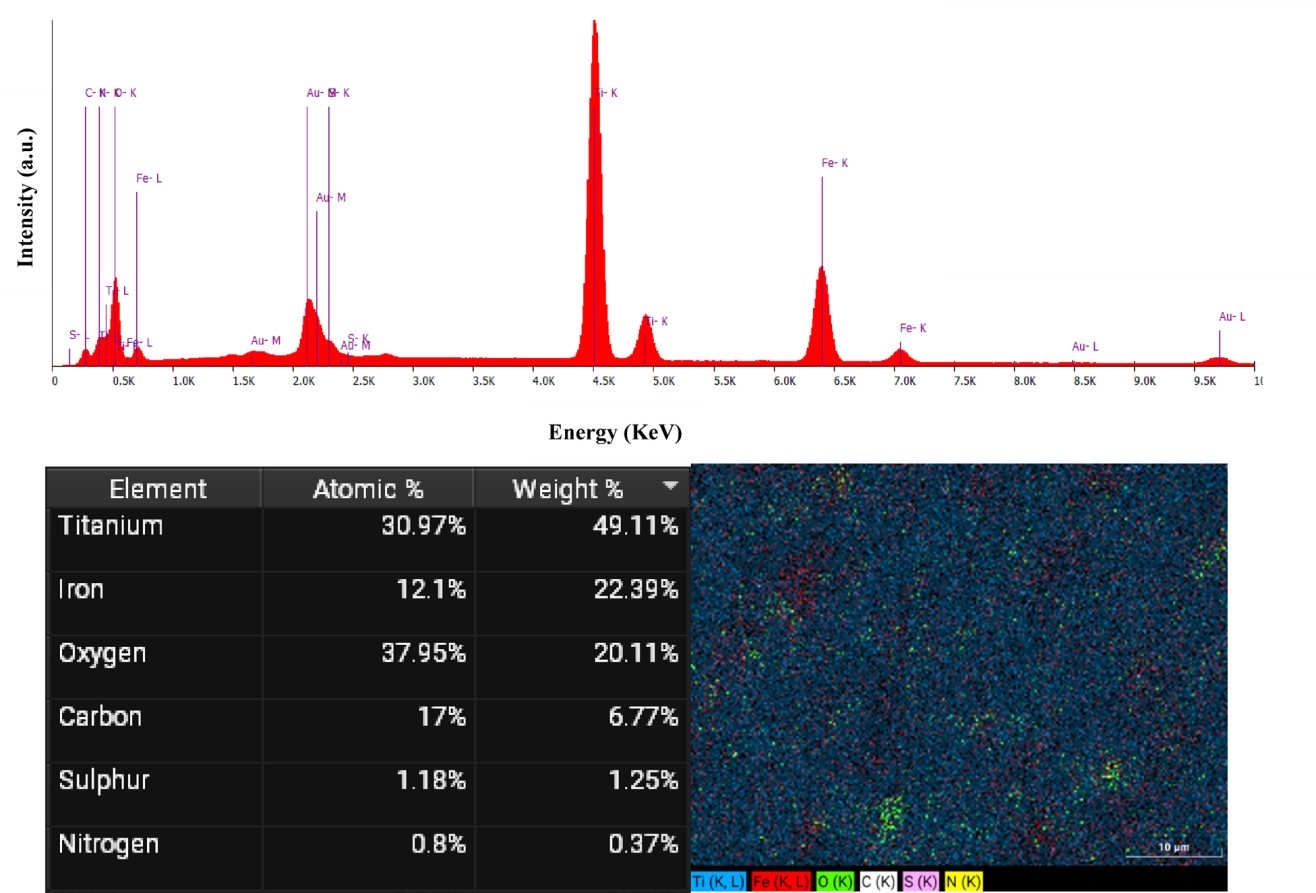
EDS pattern and the corresponding elemental mapping images of Fe_3_O_4_@TiO_2_–diamine–SO_3_H.

The EDS spectrum and elemental mapping of Fe_3_O_4_@TiO_2_–diamine–SO_3_H are shown in Fig. 3. The characteristic peaks of Ti, Fe, O, C, S, and N are clearly detected, confirming the coexistence of all expected elements in the composite structure. Quantitative analysis revealed that titanium (30.97 at%, 49.11 wt%) and oxygen (37.95 at%, 20.11 wt%) are dominant, consistent with the TiO_2_ shell composition, while iron (12.1 at%, 22.39 wt%) indicates the presence of the Fe_3_O_4_ magnetic core. Additionally, carbon (17 at%, 6.77 wt%) and nitrogen (0.8 at%, 0.37 wt%) originate from the diamine functional groups, and the detection of sulfur (1.18 at%, 1.25 wt%) verifies the successful incorporation of sulfonic acid moieties on the catalyst surface. The uniform elemental distribution observed in the mapping images further confirms the homogeneous dispersion of Ti, Fe, O, C, N, and S across the composite, indicating that the functionalization process was successful and well-distributed. The results substantiate the structural stability and effective surface functionalization of the synthesized Fe_3_O_4_@TiO_2_–diamine–SO_3_H catalyst.

**Fig. 4:**
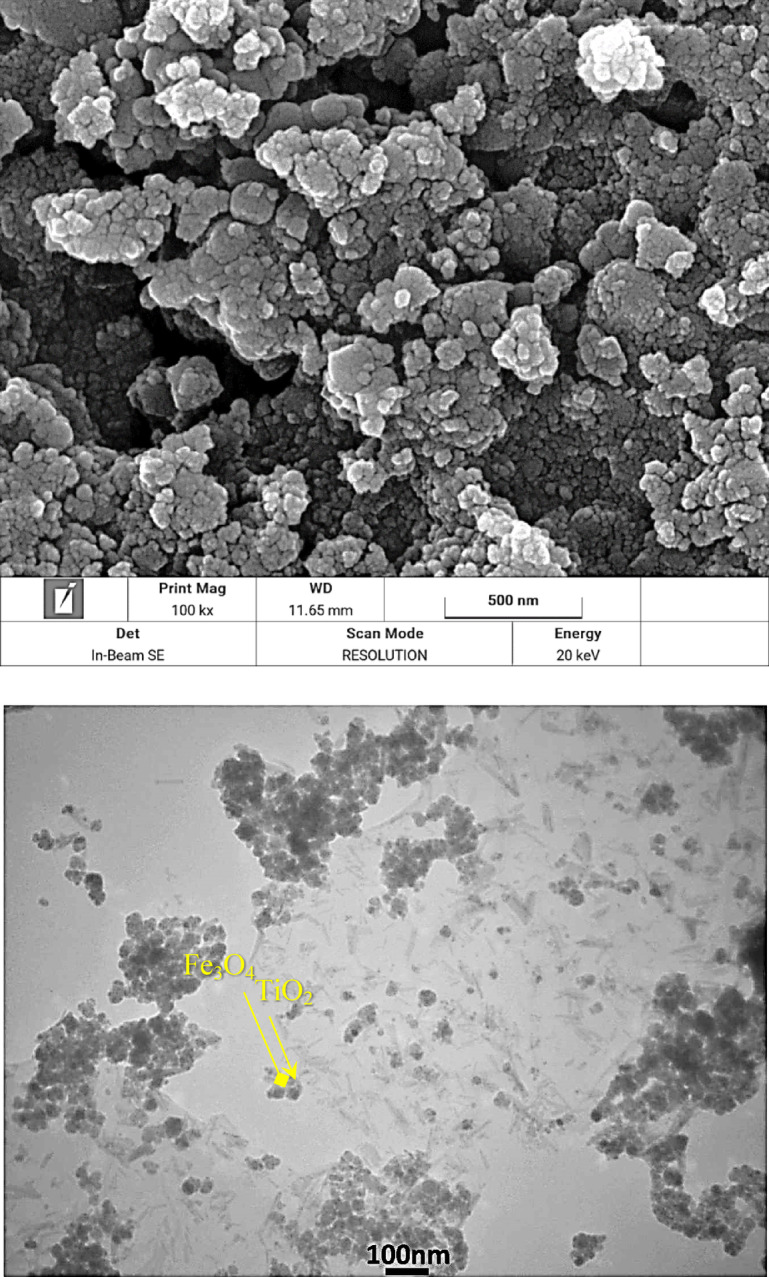
FESEM, and TEM images of Fe_3_O_4_@TiO_2_-diamine-SO_3_H.

FESEM, and TEM images (Fig. 4) reveal quasi-spherical, mildly aggregated particles with a narrow size dispersion. In the FE-SEM images, the Fe_3_O_4_@TiO_2_–diamine–SO_3_H sample exhibits a rough and wrinkled surface morphology, which is consistent with the conformal TiO₂ coating followed by subsequent organic (diamine) and –SO₃H functionalization. Primary particle diameters fall in the sub-200 nm regime, while the soft aggregation into clusters is attributed to magnetic dipole–dipole interactions. The uniform contrast and clean boundaries between particles indicate good morphological integrity without sintering or plate-like impurities. Also, TEM micrographs clearly display a core–shell architecture in which a darker Fe_3_O_4_ core is enveloped by a lighter TiO_2_ shell. Overall, FESEM and TEM verify the successful formation of a well-defined Fe_3_O_4_ core with a continuous TiO_2_ shell and stable surface functional layer. These observations are in agreement with the elemental mapping (Fig. 3), which shows homogeneous distributions of Ti and Fe throughout the composite.

**Fig. 5:**
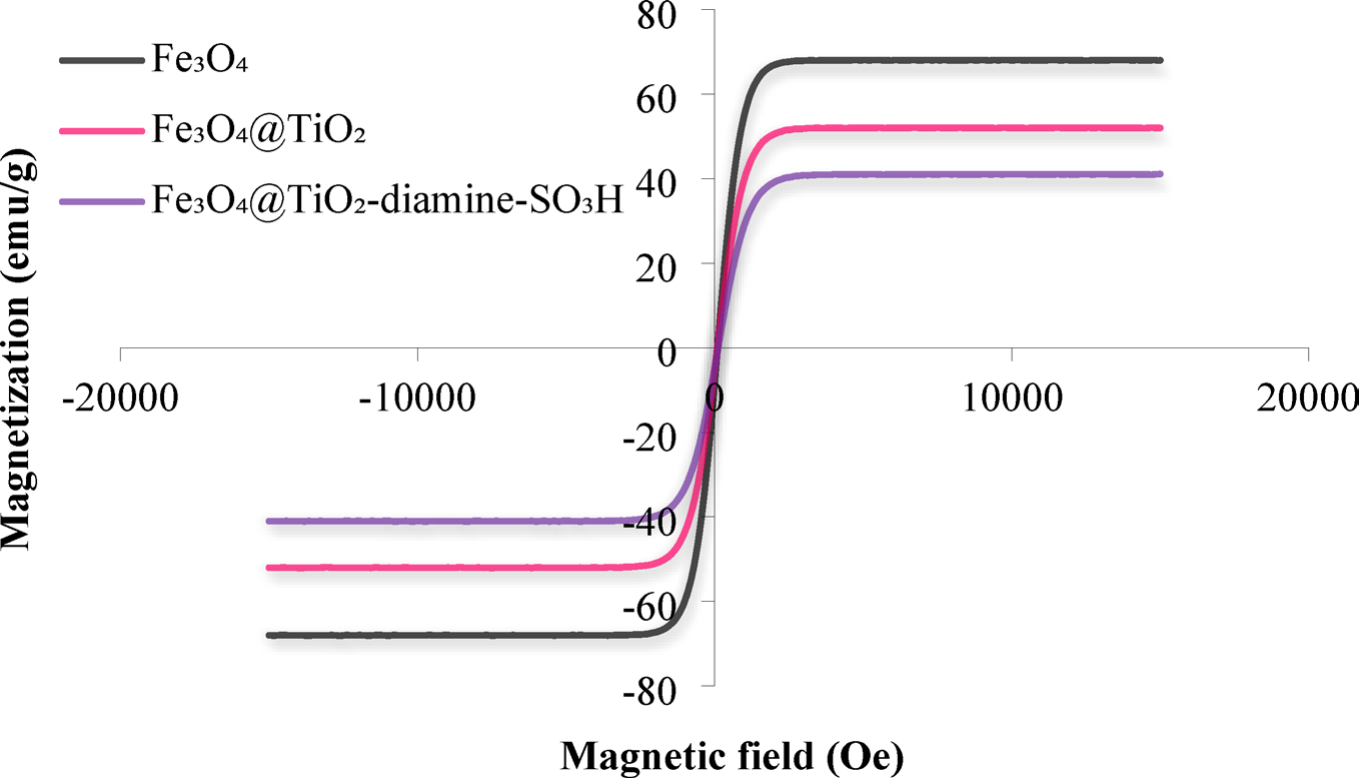
Magnetization curves of Fe_3_O_4_, Fe_3_O_4_@TiO_2_, and Fe_3_O_4_@TiO_2_-diamine-SO_3_H.

As shown in Fig. 5, pristine Fe_3_O_4_ nanoparticles exhibited a high saturation magnetization (Ms ≈ 68 emu·g^−1^) and low coercivity (Hc ≈ 0.10 kOe), confirming their superparamagnetic nature. After TiO_2_ coating, Ms decreased to ≈ 52 emu·g^−1^ while maintaining low coercivity, which can be attributed to the dilution of the magnetic phase by the non-magnetic TiO_2_ shell and a slight increase in surface anisotropy. Similar trends have been reported in previous studies, where TiO_2_ or SiO₂ coated Fe_3_O_4_ nanoparticles exhibited a significant reduction in Ms while preserving soft magnetic behavior due to the non-magnetic coating layer^83^. Furthermore, surface functionalization with diamine and sulfonic acid (–SO_3_H) groups led to an additional decrease in Ms (≈ 41 emu·g^−1^) and a slight increase in Hc (≈ 0.12 kOe), which is ascribed to the formation of an organic shell and surface spin disorder induced by chemical grafting. Similar effects of organic modification on magnetic properties have been demonstrated in sulfonic acid–functionalized Fe_3_O_4_ nanocatalysts, where the attachment of non-magnetic organic moieties reduced Ms due to surface spin canting and coverage by diamagnetic groups^84^. These consistent observations confirm that the synthesized Fe_3_O_4_@TiO_2_–diamine–SO_3_H nanostructure retains soft magnetic behavior with negligible remanence, ensuring rapid magnetic separation during catalytic applications.

**Fig. 6:**
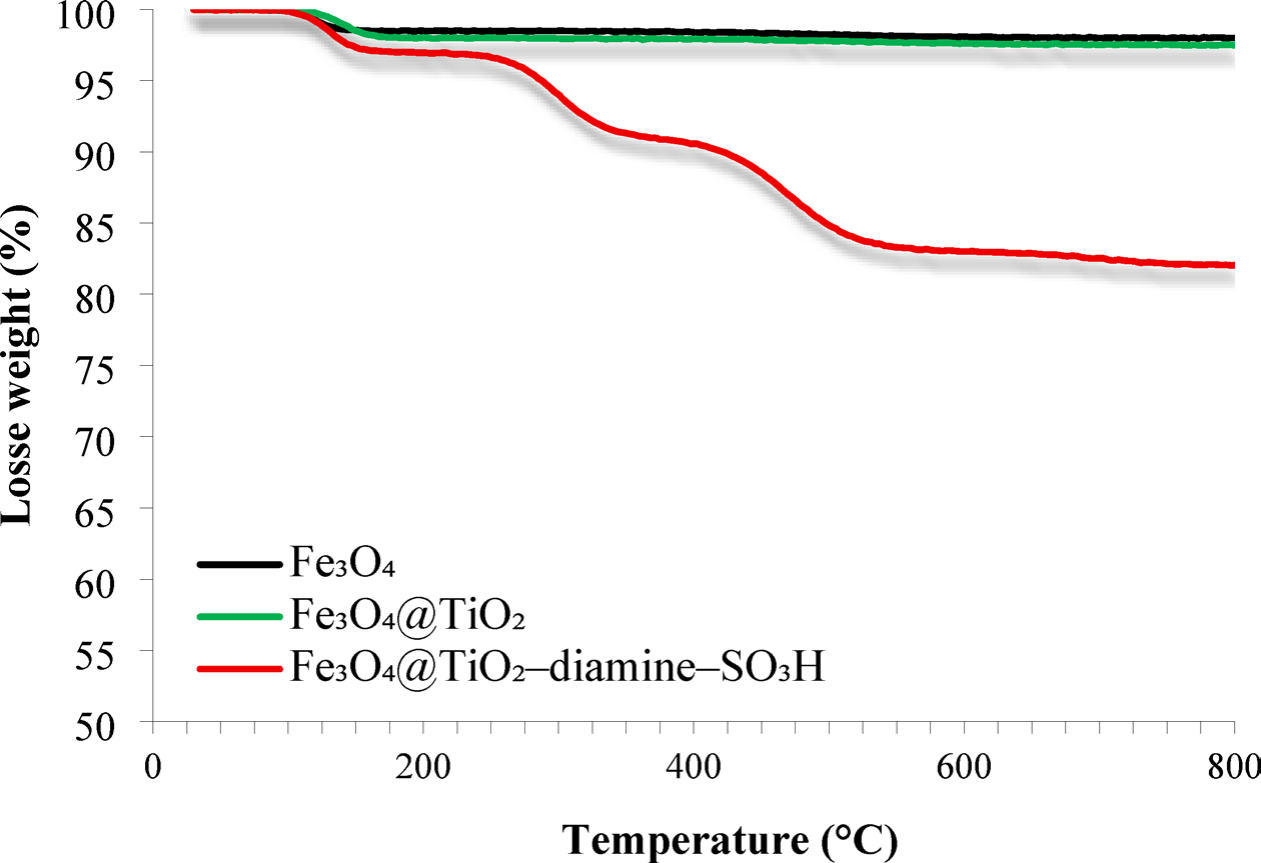
TGA curves of Fe_3_O_4_, Fe_3_O_4_@TiO_2_, and Fe_3_O_4_@TiO_2_-diamine-SO_3_H.

The thermal stability and compositional differences of Fe_3_O_4_, Fe_3_O_4_@TiO_2_, and Fe_3_O_4_@TiO_2_–diamine–SO_3_H were evaluated by TGA in the range of 25–800 °C (Fig. 6). As shown in Fig. 6, the pristine Fe_3_O_4_ shows negligible mass loss (~ 2%), mainly due to moisture removal, confirming its high thermal stability. After TiO₂ coating, a slight increase (~ 2.5%) is observed owing to surface hydroxyl groups, consistent with previous reports on Fe_3_O_4_@TiO_2_ nanocomposites^85^. The Fe_3_O_4_@TiO_2_-diamine–SO_3_H exhibits multi-step degradation (~ 18%) between 130 and 580 °C, attributed to decomposition of the diamine linker and -SO_3_H groups, similar to other studies on organically functionalized magnetic nanoparticles^86^. These results clearly demonstrate progressive incorporation of inorganic and organic layers while maintaining core stability.

**Fig. 7:**
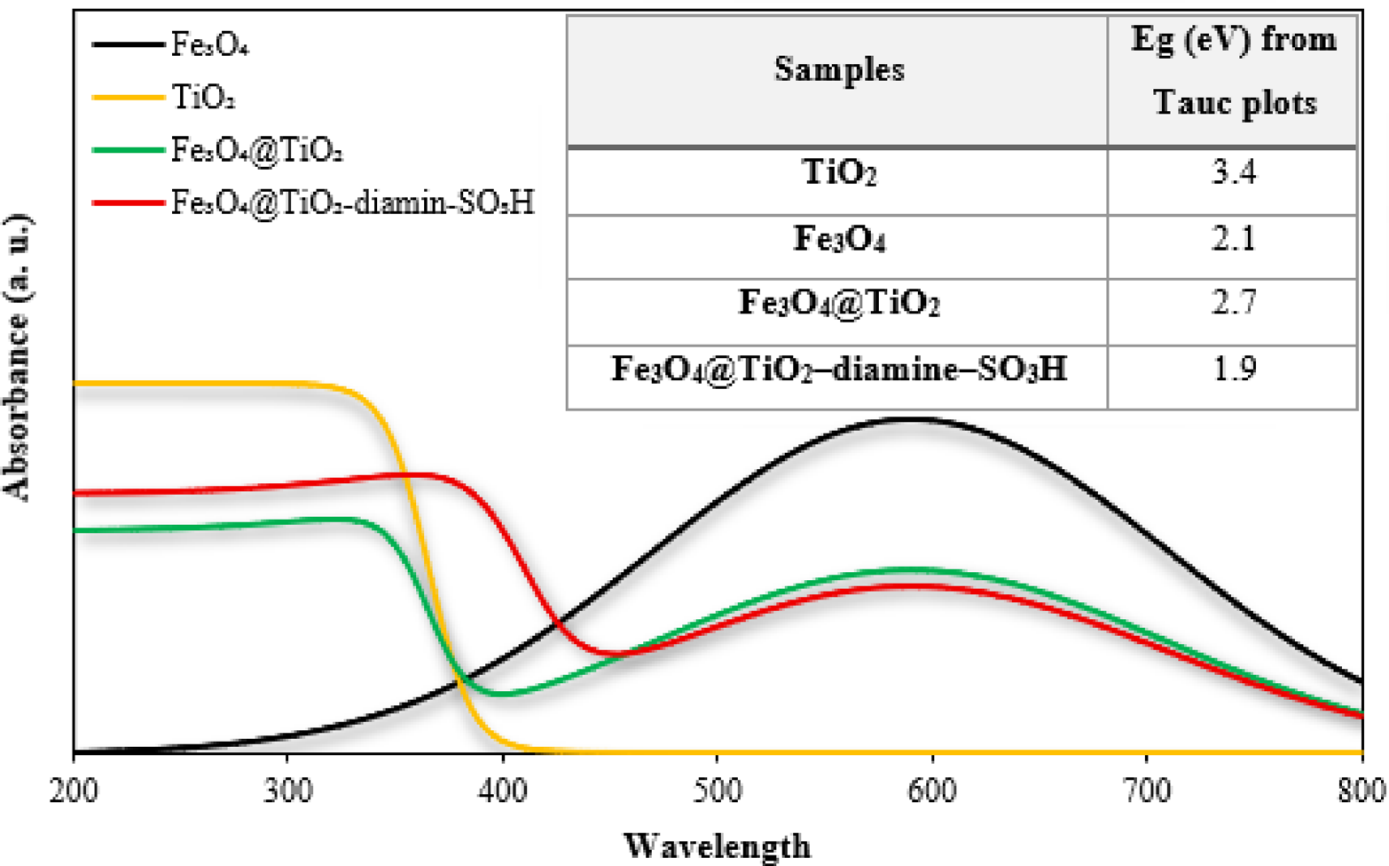
UV-Vis absorption spectra, and Optical band gap energies (Eg) of Fe_3_O_4_, TiO_2_, Fe_3_O_4_@TiO_2_, and Fe_3_O_4_@TiO_2_-diamine-SO_3_H.

The optical absorption properties of Fe_3_O_4_, TiO_2_, Fe_3_O_4_@TiO_2_, and Fe_3_O_4_@TiO_2_–diamine–SO_3_H were investigated by UV–Vis spectroscopy in the range of 200–800 nm, and the corresponding Tauc plots were employed to estimate their optical band gaps (Fig. 7). As seen in Fig. 7, pure TiO_2_ exhibits an absorption edge around ~ 335 nm, giving an optical band gap ~ 3.4 eV, consistent with the anatase phase reported in prior works. Fe_3_O_4_ alone shows broad visible absorption with an onset near ~ 590 nm, corresponding to ~ 2.1 eV, similar to values reported for Fe_3_O_4_ in magnetite-based composites^87^. In the Fe_3_O_4_@TiO_2_ core–shell, the absorption features of both components overlap, and the Tauc plot indicates a narrower band gap than pure TiO_2_, evidencing interfacial coupling and enhanced visible light response^88^. After functionalization (Fe_3_O_4_@TiO_2_–diamine–SO_3_H), an additional red shift and further narrowing (≈ 1.9 eV) is observed, which we attribute to the introduction of localized states by –SO_3_H groups and enhanced charge transfer pathways, a behavior observed in other multicomponent photocatalysts. Overall, these optical results confirm that stepwise modification of TiO_2_ with Fe_3_O_4_ and subsequent functionalization not only preserved the anatase crystalline phase but also effectively extended the absorption edge into the visible region.

### Catalyst evaluation for EMF production from HMF

**Fig. 8:**
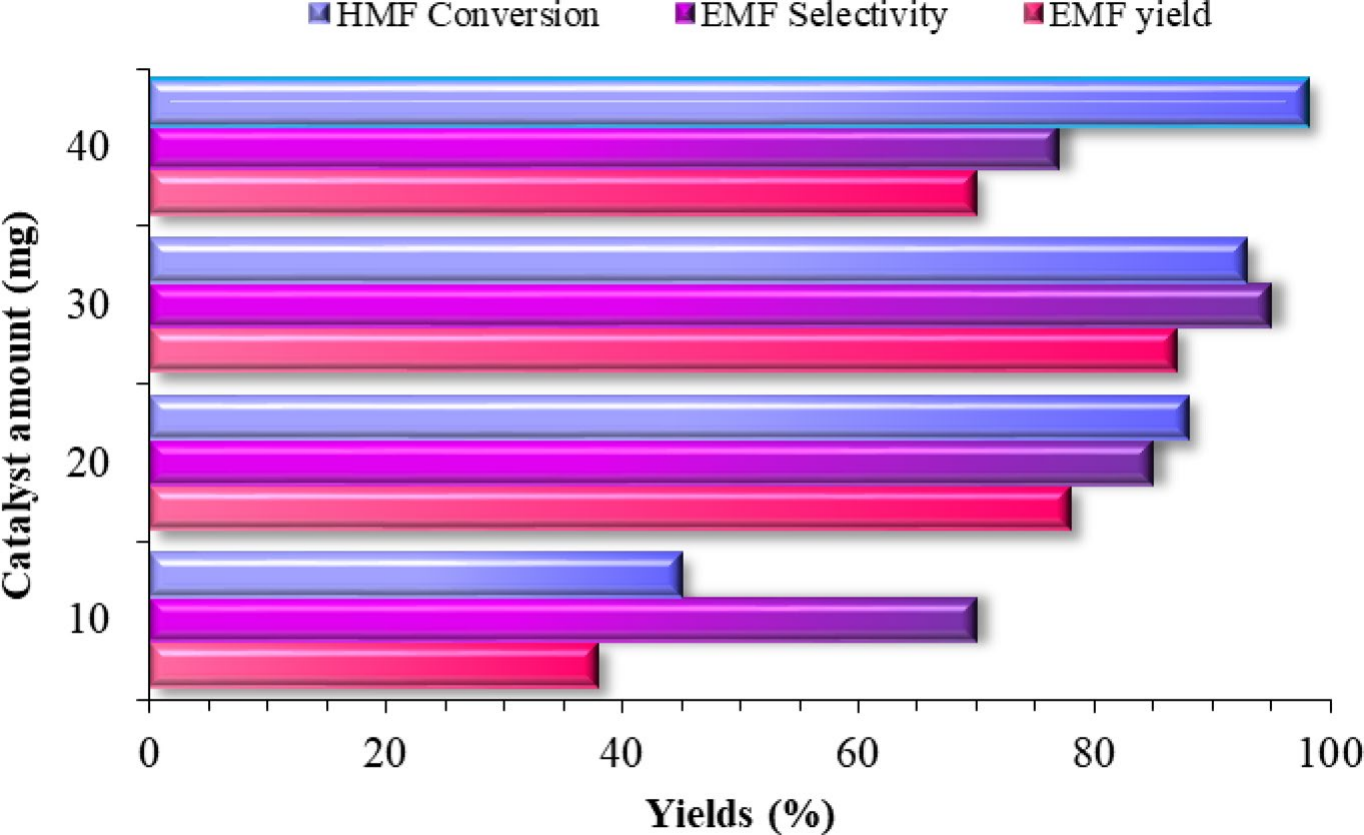
Effect of catalyst amount in etherification of HMF over Fe_3_O_4_@TiO_2_-diamine-SO_3_H, (Reaction conditions: HMF (1 mmol), EtOH (4 mL), Catalyst, White LED (15 W), 100 °C, 60 min).

**Fig. 9:**
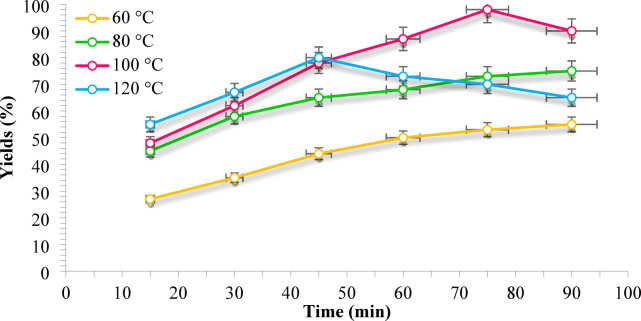
Effect of ration temperature and time in etherification of HMF over Fe_3_O_4_@TiO_2_-diamine-SO_3_H, (Reaction conditions: HMF (1 mmol), EtOH (4 mL), Catalyst (30 mg), White LED (15 W)).

**Fig. 10:**
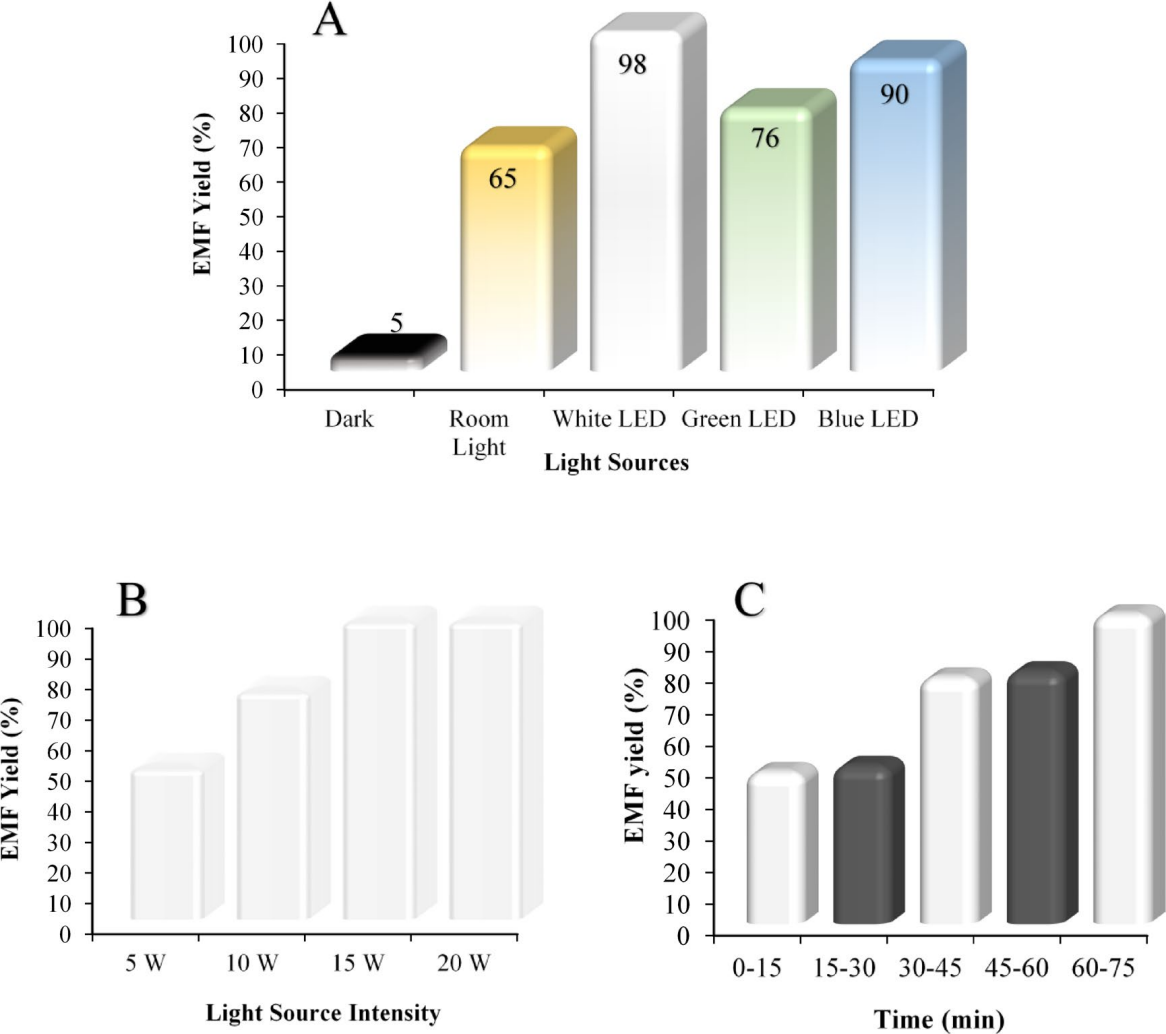
Effects of **(A)** different light sources (Reaction conditions: HMF (1 mmol), EtOH (4 mL), Catalyst (30 mg), LED (15 W), 100 °C, 75 min)), **(B)** white LED light intensity (Reaction conditions: HMF (1 mmol), EtOH (4 mL), Catalyst (30 mg), white LED (15 W), 100 °C, 75 min) on the conversion of HMF to EMF, and **(C)** Comparison of light − dark cycles with continuous exposure to white LED light (Reaction conditions: HMF (1 mmol), EtOH (4 mL), Catalyst (30 mg), white LED (15 W), 100 °C, 75 min).

 After the successful synthesis and characterization of the Fe_3_O_4_@TiO_2_–diamine–SO_3_H catalyst, its catalytic performance was systematically evaluated for the conversion of HMF to EMF under visible light irradiation. To investigate the influence of acid site concentration, catalysts with varying ClSO_3_H loadings were synthesized and tested (Table S1, Supplementary Material). The results revealed that the highest EMF yield was obtained with the catalyst containing 1.41 mmol g⁻¹ of –SO_3_H groups. Further increasing the acid density beyond this value led to a decrease in yield, which can be attributed to the promotion of side reactions and partial degradation of HMF under firm acidity. In addition, the effects of catalyst dosage, reaction temperature, reaction time, light source, and light intensity were systematically optimized, and the corresponding results are presented in Figs. 8, 9 and 10. Collectively, these findings highlight the crucial role of –SO_3_H loading and photocatalytic contribution of the TiO_2_ shell in achieving superior catalytic efficiency compared with unmodified control materials.

Subsequently, the catalyst dosage had a critical influence on the etherification of HMF to EMF. As illustrated in Fig. 8, elevating the catalyst loading from 10 to 40 mg drove HMF conversion from 45% to 98%, a result of the increased density of accessible acidic sites. An optimal catalyst mass of 30 mg, however, afforded the peak EMF yield and selectivity of 87% and 95%, respectively, suggesting a balance between activity and potential side reactions at higher loadings. Increasing the catalyst dosage beyond this value led to a decline in both EMF yield and selectivity, likely due to excessive acidic sites promoting side reactions and byproduct formation. Therefore, 30 mg was identified as the optimal catalyst dosage for EMF production. Furthermore, to verify the role of SO₃H groups in this reaction system, control experiments were conducted under identical conditions without catalyst (blank reaction) and in the presence of Fe_3_O_4_, TiO_2_, Fe_3_O_4_@TiO_2_, and Fe_3_O_4_@TiO_2_–diamine. Only trace amounts of EMF were obtained even after 6 h of heating, confirming the crucial role of SO₃H groups in driving the reaction.

The reaction temperature and time play pivotal roles in governing the efficiency of HMF etherification to EMF, as they directly influence both the reaction kinetics and the stability of the desired product. To further optimize the process, a systematic investigation was conducted to evaluate the effect of reaction temperature (60–120 °C) and reaction time (15–150 min) in ethanol as the solvent (Fig. 9). As illustrated, a gradual increase in temperature from 60 °C to 100 °C resulted in a pronounced enhancement in EMF yield, with the maximum yield of approximately 98% being achieved at 100 °C after 75 min. This improvement can be attributed to the higher kinetic energy at elevated temperatures, which accelerates the etherification rate. However, when the temperature was further raised to 120 °C, although an initially high yield was observed, a sharp decline occurred beyond 45 min. This decline is likely due to the hydrolytic degradation of both HMF and EMF under harsher thermal conditions, as also reported in previous studies^89^. Similarly, while extending the reaction time at moderate temperatures favored HMF conversion, excessive durations especially at higher temperatures led to a continuous decrease in EMF yield. This is primarily because prolonged heating accelerates side reactions, promoting the formation of ethyl levulinate (EL) and insoluble humins from EMF^89^. Therefore, by balancing reaction kinetics with product stability, the optimal conditions for efficient EMF synthesis were established at 100 °C and 75 min, ensuring a high yield while minimizing by-product formation.

The Fig. 10 investigates the effect of different light sources (Fig. 10A), light intensity (Fig. 10B), and light-dark cycles (Fig. 10C). Figure 10A shows that various light sources have a significant impact on the reaction yield. The best yield is obtained with white LED light, which provides a 98% yield, followed closely by blue LED light, which yields 90%. In contrast, the reaction yield under room light (65%) and in darkness (5%) is much lower. This suggests that specific light sources, especially white LED light, have a favorable spectrum that can effectively activate photochemical and catalytic processes, leading to improved reaction yields. Figure 10B illustrates the effect of light intensity on the reaction yield. As shown, increasing the light intensity from 5 W to 15 W significantly enhances the reaction yield. At 5 W, the yield is approximately 50%, which increases to 75% at 10 W and reaches a maximum of 98% at 15 W.

This upward trend indicates that higher light intensity promotes photocatalytic activity, likely by generating more photoinduced charge carriers (electrons and holes), which accelerate the reaction. However, a further increase in light intensity from 15 W to 20 W does not lead to any additional improvement in yield, which remains constant at 98%. This plateau suggests that the system has reached a saturation point, possibly due to the limited number of active catalytic sites or the maximum absorption capacity of the photocatalyst. Therefore, it can be concluded that while light intensity plays a critical role in enhancing the reaction up to a certain level, beyond that point, additional light energy does not contribute to higher yields. Finally, Fig. 10C, which compares light-dark cycles, shows that alternating between light exposure (0–15 min; 30–45 min; 60–75 min) and periods of darkness (15–30 min; 45–60 min) significantly enhances the reaction yield. This cyclical exposure to light may allow the catalyst to regenerate or be more effectively activated during the light phases. In contrast, the dark phases might provide time for stabilization or additional activation. This process is crucial for systems where catalyst regeneration is crucial, and it can help maintain or even improve catalytic efficiency over time.

### Catalyst evaluation in direct EMF synthesis from carbohydrates

**Fig. 11:**
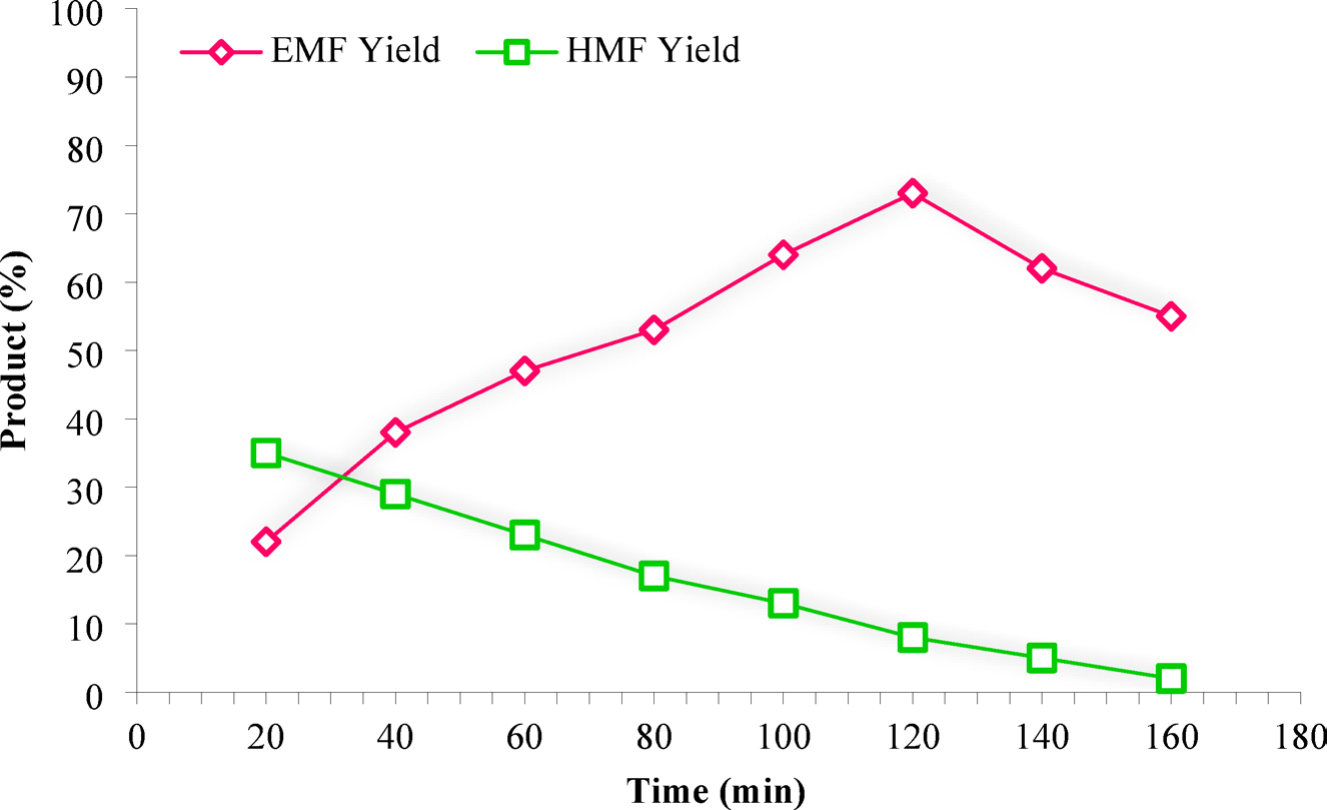
Conversion of fructose (via HMF intermediate) and direct transformation of HMF to EMF over Fe_3_O_4_@TiO_2_-diamine-SO_3_H, (Reaction conditions: fructose (1 mmol), EtOH (4 mL), Catalyst (30 mg), white LED (15 W), 100 °C).

Given the economic constraints associated with HMF as a feedstock, fructose represents a more accessible and cost-effective substrate for EMF production. When fructose or biomass-derived carbohydrates were used as substrates, the reaction proceeded via in situ dehydration to HMF, followed by etherification to EMF. In contrast, when pure HMF was employed as the feed, a direct conversion to EMF occurred under identical conditions. This clarifies that both pathways (fructose → HMF → EMF and HMF → EMF) were explored and compared in this study. Building on the successful etherification of HMF using Fe_3_O_4_@TiO_2_-diamine-SO_3_H, we explored the direct conversion of fructose into EMF via sequential dehydration and etherification under the same optimized conditions (30 mg catalyst, white LED 15 W, 100 °C, 75 min). As shown in Fig. 11, the EMF formation from fructose proceeded at a slower rate compared to HMF, achieving a maximum yield of 73% after 120 min. Prolonging the reaction to 160 min caused the yield to drop to 55%, primarily due to side reactions converting EMF into ethyl levulinate (EL) and humins. This behavior is further explained by the role of HMF as a reactive intermediate; water generated during fructose dehydration can interfere with etherification, limiting EMF formation^40^.

As shown in Figs. 9 and 11, prolonging the reaction time leads to a gradual decrease in EMF yield, which can be attributed to secondary degradation of EMF under strongly acidic and high-temperature conditions. Therefore, the maximal yield observed (98% from HMF and 73% from fructose) results from a balance between the formation of EMF from HMF and its subsequent degradation at longer reaction times. To verify the accuracy of the reported yield, the product distribution was analyzed by HPLC using authentic standards of HMF and EMF, and the quantification was performed based on calibration curves.

### Control experiments

 To assess the individual contribution of the magnetic core and the photocatalytic TiO_2_ shell, control experiments were performed under the same reaction conditions used for catalytic tests. The control experiments (Table 1) clearly demonstrate the critical role of the –SO₃H functional groups in achieving high EMF yields. In a typical control run, HMF (1 mmol) and EtOH (4 mL) were placed in a 25 mL sealed vessel, the catalyst (30 mg) was added, and the mixture was heated to 100 °C under stirring and simultaneous illumination by a 15 W white LED for 75 min. After reaction, the mixture was cooled, the solid was separated magnetically (or by filtration for non-magnetic TiO_2_), and the liquid phase was analyzed by HPLC as described in Sect. “HPLC analysis”. Blank experiments (no solid) were also carried out under identical conditions. Each experiment was repeated at least twice and the reported yield values represent the average of independent runs. The reported selectivity and yields were calculated as described in the Supplementary Material. In the absence of any catalyst, no EMF formation was detected, confirming that neither of these materials alone is catalytically active for etherification. The control experiments shown in Table 1 confirm that neither TiO_2_ nor Fe_3_O_4_ alone promotes significant conversion of HMF to EMF under the applied conditions. The Fe_3_O_4_@TiO_2_ core–shell composite without further modification afforded only a trace amount of EMF, indicating that the photocatalytic contribution of TiO_2_ by itself is insufficient to drive the reaction under the studied conditions. Upon functionalization with diamine linkers (Fe_3_O_4_@TiO_2_–diamine), a moderate yield of 15% EMF was obtained, suggesting that the organic functional layer improves interaction with HMF but still lacks the required acidity for efficient etherification. In contrast, the fully functionalized Fe_3_O_4_@TiO_2_–diamine–SO_3_H catalyst exhibited outstanding activity, delivering 98% yield of EMF. These observations confirm that the synergistic combination of strong Brønsted acid sites (–SO_3_H groups) with the photocatalytic properties of TiO_2_ and the magnetic recoverability of Fe_3_O_4_ is responsible for the superior catalytic performance of the developed system.

**Table 1:** Control experiments for the etherification of HMF to EMF.

Entry	Catalyst	EMF Yield (%)
**1**	-	-
**2**	TiO_2_	-
**3**	Fe_3_O_4_	-
**4**	Fe_3_O_4_@TiO_2_	Trace
**5**	Fe_3_O_4_@TiO_2_-diamine	15
**6**	Fe_3_O_4_@TiO_2_-diamine-SO_3_H	98

(Reaction conditions: HMF: 1 mmol, EtOH: 4 mL, catalyst: 30 mg, white LED: 15 W, 100 °C, 75 min).

 The design of the Fe_3_O_4_@TiO_2_–diamine–SO_3_H catalyst combines multiple functional components. Fe_3_O_4_ provides superparamagnetic properties, which enable rapid magnetic separation and recycling of the catalyst, while also contributing to charge-transfer processes through Fe²⁺/Fe³⁺ redox cycling. The TiO_2_ shell serves as a stable photocatalyst that generates photogenerated electron–hole pairs under white-LED illumination, thereby enhancing substrate activation and synergistically accelerating the acid-catalyzed etherification process. Although TiO_2_ can potentially generate highly reactive oxygen species under irradiation, which may cause product degradation, the optimized reaction conditions (moderate light intensity, controlled reaction time) effectively suppress over-oxidation. Furthermore, recycling experiments demonstrate that the catalyst maintains high activity and structural integrity over multiple runs, confirming that no significant degradation of the catalyst occurs. Control experiments (Table 1) further verify that neither Fe_3_O_4_ nor TiO_2_ alone exhibited catalytic activity, and that the superior performance arises from the synergistic combination with surface –SO₃H groups.

 To further examine the applicability of the catalyst under higher substrate loadings, additional reactions were carried out using 5 mmol and 10 mmol of HMF under otherwise identical conditions. As summarized in Table S2 (Supplementary Material file), the EMF yield decreased from 97% (1 mmol HMF) to 82% (5 mmol HMF) and 68% (10 mmol HMF), mainly due to light penetration and mass-transfer limitations at higher concentrations. Nevertheless, the overall productivity of the catalyst increased significantly, from 4.9 g.EMF g⁻¹ catalyst (1 mmol scale) to 21.3 g EMF.g⁻¹ catalyst (5 mmol) and 35.2 g EMF.g⁻¹ catalyst (10 mmol). These results confirm that the catalyst remains active and effective at elevated loadings, although further optimization of reactor design and illumination conditions will be required for practical scale-up.

**Fig. 12:**
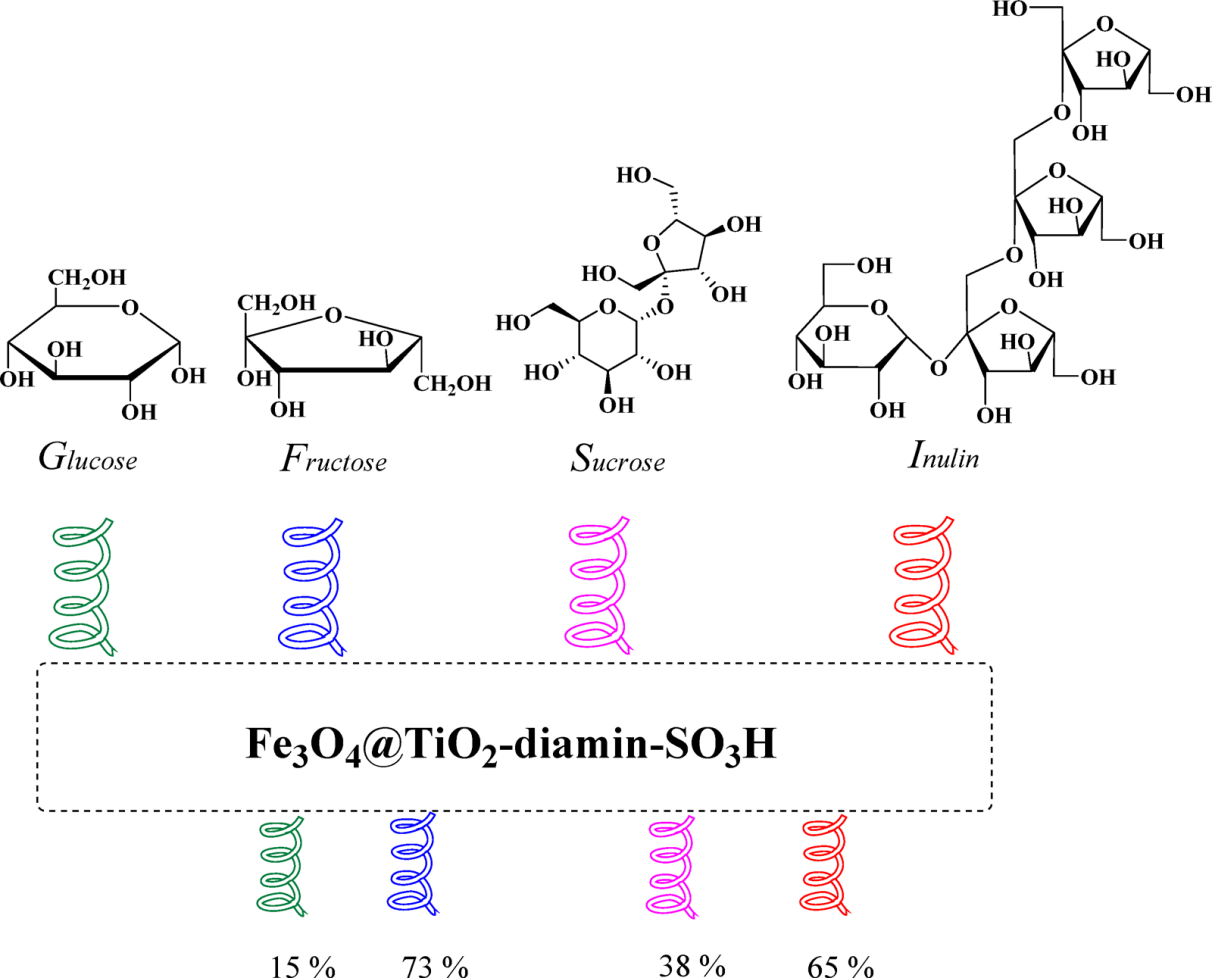
Conversion of various carbohydrates to EMF over Fe_3_O_4_@TiO_2_-diamine-SO_3_H, (Reaction conditions: Carbohydrates (1 mmol), EtOH (4 mL), Catalyst (30 mg), white LED (15 W), 100 °C).

The applicability of this catalytic system was further tested using other carbohydrates, including glucose, sucrose, and inulin (Fig. 12). Glucose and sucrose representing aldose-based substrates produced considerably lower EMF yields. In contrast, Inulin a fructose-based polysaccharide, afforded a substantial EMF yield of 65%, slightly lower than fructose due to the necessity of an additional hydrolysis step^90^. These results highlight the effectiveness of the Fe_3_O_4_@TiO_2_-diamine-SO_3_H catalyst for converting fructose-containing carbohydrates while underscoring the challenges associated with aldose substrates. Overall, this study demonstrates that both the substrate structure and reaction pathway significantly influence EMF yield, providing valuable insights for designing efficient biomass-to-biofuel conversion strategies.

### Catalyst recyclability and leaching test

**Fig. 13:**
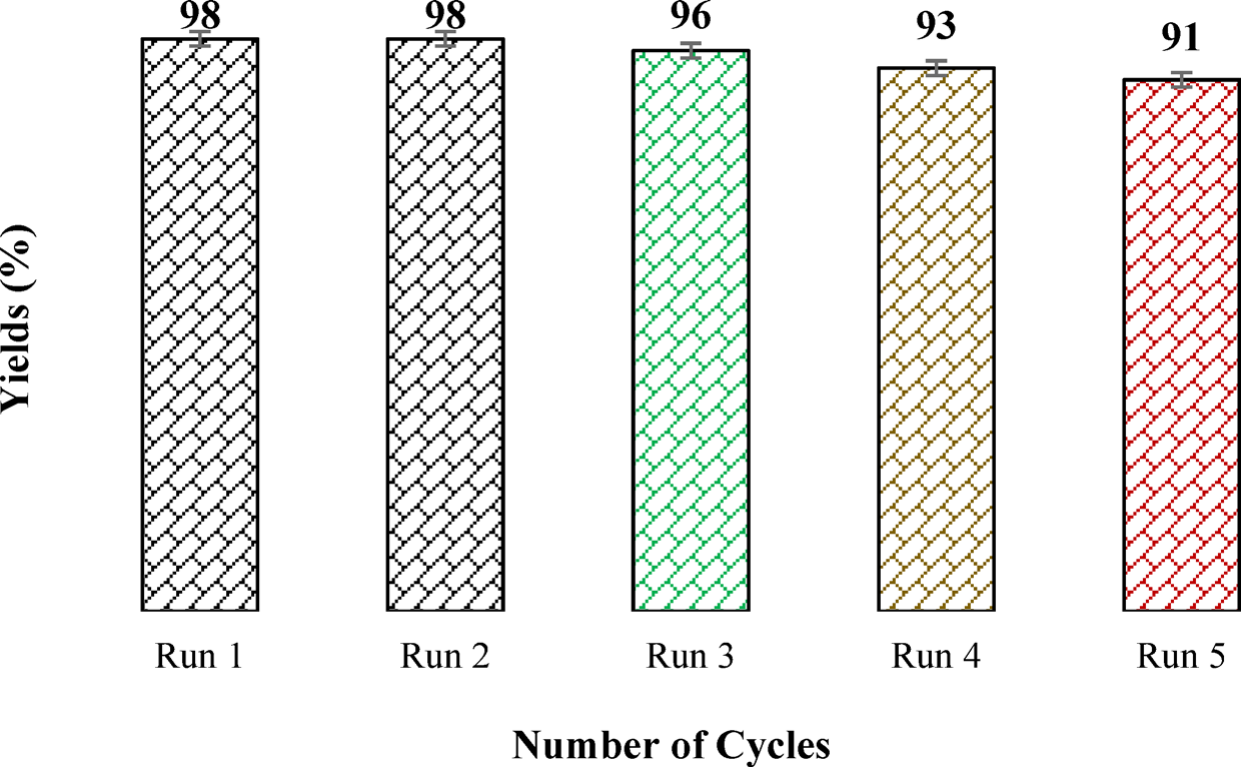
Reusability test for Fe_3_O_4_@TiO_2_-diamine-SO_3_H, (Reaction conditions: HMF (1 mmol), EtOH (4 mL), Catalyst (30 mg), white LED (15 W), 100 °C, 75 min).

Beyond high activity, excellent recyclability is essential for the practical deployment of heterogeneous catalysts. To assess this feature, the recyclability of Fe_3_O_4_@TiO_2_-diamine-SO_3_H was examined over five consecutive cycles for the etherification of HMF to EMF under optimized reaction conditions (Fig. 13). After each run, the catalyst was magnetically separated from the reaction mixture, thoroughly washed with ethanol to remove any adsorbed reagents from its surface, and dried under vacuum prior to reuse. As illustrated in Fig. 13, the EMF yield in the first cycle reached 98%, while only a slight decrease was observed after repeated use, maintaining 91% yield in the fifth cycle. Furthermore, a comparative analysis of the XRD patterns (Fig. S2) for the fresh and five-times-used catalysts indicated that the structural integrity of Fe_3_O_4_@TiO_2_-diamine-SO_3_H was well preserved. In addition, both the acidity (measured by acid–base titration) and sulfur content (determined via elemental analysis) of the spent catalyst after five cycles were nearly identical to those of the fresh material. These results collectively confirm the remarkable durability and stability of Fe_3_O_4_@TiO_2_-diamine-SO_3_H under the applied reaction conditions, demonstrating its strong potential for repeated use without significant loss of efficiency.

**Fig. 14:**
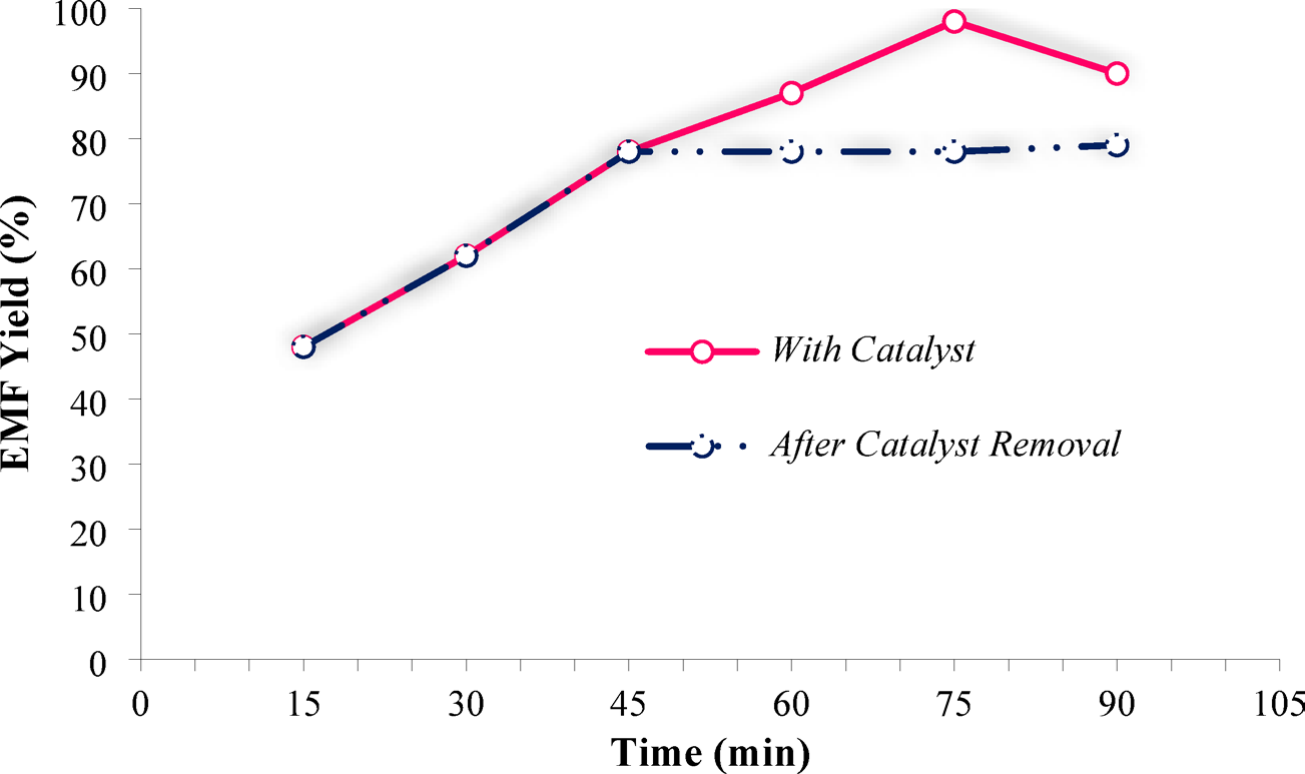
Leaching test for Fe_3_O_4_@TiO_2_-diamine-SO_3_H, (Reaction conditions: HMF (1 mmol), EtOH (4 mL), Catalyst (30 mg), white LED (15 W), 100 °C).

To verify the true heterogeneous nature of the catalytic process, a hot filtration (leaching) test was conducted under the optimized reaction conditions (Fig. 14). The reaction was initiated in the presence of Fe_3_O_4_@TiO_2_-diamine-SO_3_H. After reaching 50% conversion, the catalyst was magnetically separated from the reaction medium while the mixture was maintained at the reaction temperature. The filtrate was then allowed to react further under identical conditions without any solid catalyst. No additional increase in EMF yield was observed after catalyst removal, indicating that no catalytically active species had leached into the reaction medium. Furthermore, ICP–OES analysis of the filtrate revealed negligible concentrations of Fe and S, confirming that the metallic and acidic active sites were firmly anchored on the catalyst surface and not released into the solution during the reaction. These results clearly demonstrate that the etherification of HMF to EMF proceeds through a purely heterogeneous pathway, and the excellent reusability of Fe_3_O_4_@TiO_2_-diamine-SO_3_H is not compromised by the leaching of active components.

**Scheme 3:**
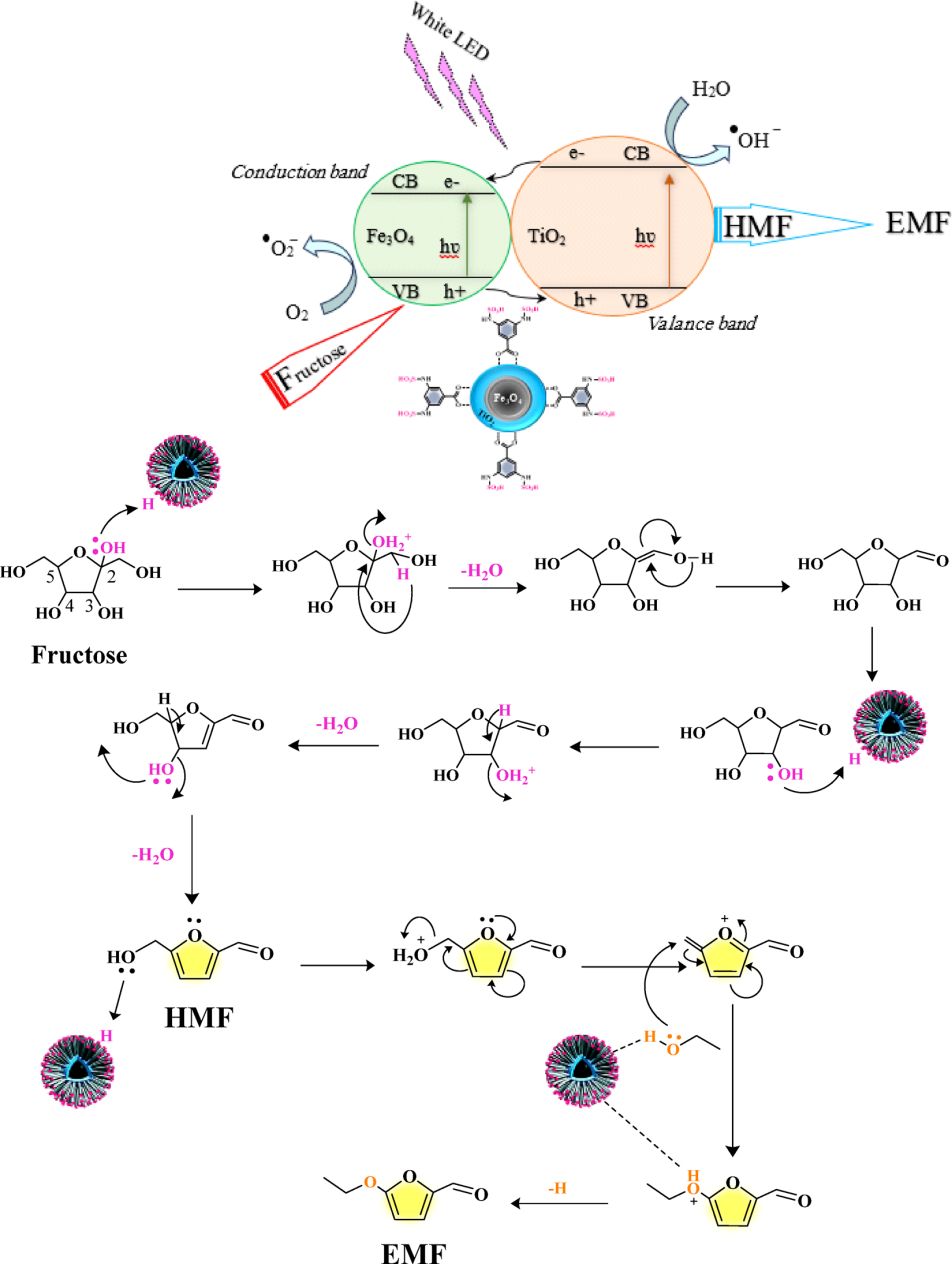
Proposed mechanism for fructose→HMF→EMF over Fe_3_O_4_@TiO_2_-diamine-SO_3_H.

The proposed mechanism for the catalytic transformation of fructose into EMF over Fe_3_O_4_@TiO_2_–diamine–SO₃H under white-LED irradiation is illustrated in Scheme 3^91^. This process proceeds through two main consecutive stages: (i) dehydration of fructose to HMF, and (ii) photo-assisted etherification of HMF with ethanol to afford EMF. In the first stage, fructose undergoes acid-catalyzed dehydration facilitated by the Brønsted acidic –SO₃H sites on the catalyst surface. Protonation of the hydroxyl groups leads to the elimination of water molecules and the formation of an intermediate carbocation, which subsequently rearranges to yield HMF. The –SO₃H groups play a vital role in promoting these dehydration steps through proton donation and stabilization of intermediate species. In the second stage, the conversion of HMF to EMF occurs mainly via an acid-catalyzed etherification pathway, while the TiO_2_/Fe_3_O_4_ composite provides a photo-assisted enhancement under visible-light irradiation. Upon exposure to white-LED light, TiO_2_ absorbs photons and generates electron–hole (e⁻/h⁺) pairs. The photogenerated holes (h⁺) at the valence band can enhance the polarization of the C–OH bond in the hydroxymethyl group of HMF, facilitating its protonation and dehydration to a carbocation intermediate. Subsequently, ethanol acts as a nucleophile, attacking the carbocation to form the ethoxymethyl intermediate, which after deprotonation yields the target product EMF. Meanwhile, electrons (e⁻) in the conduction band of TiO_2_ can be transferred to Fe_3_O_4_, reducing oxygen to reactive oxygen species such as superoxide (O₂^•^⁻) and hydroxyl radicals (^•^OH). These species may participate in minor radical side reactions, but under the optimized conditions they mainly contribute to maintaining charge separation and preventing recombination of photogenerated carriers. This synergy between TiO_2_ and Fe_3_O_4_ effectively improves the reaction kinetics and selectivity. Recycling and FT-IR analyses of the reused catalyst confirmed that the characteristic bands of the –SO₃H groups remained unchanged after several cycles, indicating that the surface acid-site density was not affected by light irradiation (Fig. S2 B). Therefore, the overall mechanism can be described as a cooperative photo-assisted Brønsted acid catalysis, where the acid sites drive the etherification process and the TiO_2_/Fe_3_O_4_ heterostructure facilitates charge separation and photoactivation of the substrate.

¿

A detailed comparison of the catalytic efficiency of the prepared Fe_3_O_4_@TiO_2_-diamine-SO_3_H with various heterogeneous systems reported in the literature for EMF production is summarized in Table 2. The results clearly indicate that the present catalyst achieves efficient conversion of HMF and fructose to EMF under milder conditions requiring lower reaction temperatures and/or shorter reaction times than many of the previously described systems. Additionally, the magnetic nature of Fe_3_O_4_@TiO_2_-diamine-SO_3_H allows for straightforward separation from the reaction mixture using an external magnet, eliminating the need for complex purification steps.

**Table 2:** Performance of various heterogeneous catalysts in the conversion of HMF and Fructose to EMF.

Entry	Reactants	Catalysts	Solvents	Temp. (oC)	Time (min)	EMF Yield (%)	Ref.
1	HMF	A-BCMSs-SO_3_H@PTFE	EtOH	100	72 h	65	^62^
2	HMF	HNTs-SO_3_H	DMSO	126	35	86.4	^92^
3	HMF	PDVTA-SO_3_H	EtOH	110	30	87.5	^78^
4	HMF	Pd-Ru/MXene	EtOH	120	90	98	^93^
5	HMF	S-PANI-FeVO_4_(11)	EtOH	90	6 h	85	^60^
6	HMF	HSO4/SMNPs	EtOH	120	60	94.3	^94^
7	HMF	Fe_3_O_4_@TiO_2_-diamine-SO_3_H	EtOH	100	75	98	This Work
8	Fructose	S/Cl@bm_3_Q_1_	EtOH/DMSO	140	18 h	96.4	^95^
9	Fructose	MOP-SO_3_H	EtOH	100	5 h	72.2	^96^
10	Fructose	T-CeMOF	EtOH/DMSO	130	16 h	69.6	^97^
11	Fructose	Pd-Ru/MXene	EtOH	120	150	82	^93^
12	Fructose	S-PANI-FeVO_4_(11)	EtOH	90	6 h	84	^60^
13	Fructose	HSO_4_/SMNPs	EtOH	120	2 h	68.2	^94^
14	Fructose	Fe_3_O_4_@TiO_2_-diamine-SO_3_H	EtOH	100	75	73	This Work

## Conclusion

In this study, a highly efficient and magnetically separable solid acid catalyst, Fe_3_O_4_@TiO_2_–diamine–SO_3_H, was successfully synthesized and thoroughly characterized by a suite of analytical techniques, confirming its structural integrity, surface functionalities, and firm Brønsted acidity. The catalyst was applied for the direct synthesis of EMF through the etherification of HMF with ethanol and the one-pot transformation of carbohydrates into EMF under optimized conditions (100 °C, ethanol as solvent, catalyst loading of 30 mg, and reaction time of 75 min, white LED 15 W). Under these parameters, it exhibited outstanding catalytic activity, affording 98% EMF yield in the first run and retaining 91% yield even after five consecutive cycles, highlighting its remarkable stability and reusability. Leaching tests revealed negligible loss of metal species or active sites, while post-reaction characterizations confirmed that the crystalline structure and acidic properties were well preserved, ensuring sustained catalytic performance.

## Supplementary Information

Below is the link to the electronic supplementary material.

Supplementary Material 1

## Data Availability

The datasets generated and/or analyzed during the current study are not publicly available due to privacy restrictions concerning human participants, but are available from the corresponding author on reasonable request.

## Abbreviations

EMF: 5-ethoxymethylfurfural
HMF: 5-hydroxymethylfurfural
FDCA: furan dicarboxylic acid
HMFCA: 5-hydroxymethylfuran-2-carboxylic acid
DFF: 2,5-diformylfuran, LA, levulinic acid
DMF: dimethylfuran
DHMF: dihydroxymethylfuran
5-MF: 5-methylfuraldehyde
EFE: ethylfurfuryl ether
BEMF: (2,5-bis(ethoxymethyl)furan)
SPC: sulfonated nitrogen-containing polymer
DHA: dihydroxyacetone
EL: ethyl levulinate
HIPE: FTIR, Fourier transform infrared
TEM: transmission electron microscopy
EDX: energy-dispersive X-ray spectroscopy, TGA, thermogravimetric analysis
XRD: X-ray diffraction
LED: light emitting diode
