# Supplementary material for: Magnetically recoverable solid acid photocatalyst activated by white LED for sustainable high-yield synthesis of 5-ethoxymethylfurfural from biomass
Source: Sci Rep. 2025 Nov 27;15:42537. doi: 10.1038/s41598-025-27726-y (PMC12660819; doi:10.1038/s41598-025-27726-y)
Supplement: Supplementary file 1 — Supplementary Material 1 [file 41598_2025_27726_MOESM1_ESM.docx]

**Supplementary** **Material**

**Magnetically recoverable solid acid photocatalyst activated by white LED for sustainable high-yield synthesis of 5-ethoxymethylfurfural from biomass**

Pouya Ghamari Kargar^a^, Mehdi Hosseini*^a,b^

^a^ Department of Chemistry, Faculty of Basic Sciences, Ayatollah Boroujerdi University, Boroujerd, Iran

^b^ Biosensor and Energy Research center, Ayatollah Boroujerdi University, Boroujerd, Iran.

*Corresponding author: M. Hosseini

Tel.: +98 66 42468320

Fax: +98 66 42468223

Email address: Corresponding author e-mails: [Hosseini.mih@gmail.com](mailto:Hosseini.mih@gmail.com); [Hosseini.mehdi@abru.ac.ir](mailto:Hosseini.mehdi@abru.ac.ir)

**Acid–base titration for acidity measurement**

The acidity of the Fe_3_O_4_@TiO_2_–diamine–SO_3_H catalyst was determined by acid–base titration. Typically, 0.05 g of the catalyst was suspended in 25 mL of a 2 M NaCl aqueous solution and stirred for 12 h at room temperature to exchange the protons from the –SO₃H groups into the solution. The suspension was then filtered, and the filtrate was titrated with a standardized 0.01 M NaOH solution using phenolphthalein as an indicator. The total acidity (mmol H⁺ g⁻¹ catalyst) was calculated from the volume of NaOH consumed according to the relation:

((C_NaOH_ × V_NaOH_) / W_Cat_) = Acidity (mmol/g)

where C_NaOH_ is the concentration of NaOH (mol L⁻¹), V_NaOH_ is the volume of NaOH solution consumed (L), and W_Cat_ is the weight of catalyst (g).

**Table S1.** Etherification of HMF over various catalysts.

| Amount of  Fe_3_O_4_@TiO_2_-diamin-SO_3_H | Amount of  ClSO_3_H | Acidity  (mmol/g)^a^ | Yield of EMF  (%) |
| --- | --- | --- | --- |
| 1 g | 2 mmol | 0.95 | 25 |
| 1 g | 4 mmol | 1.41 | 78 |
| 1 g | 6 mmol | 1.77 | 33 |

Reaction conditions: HMF (1mmol), ethanol (4 mL), catalyst (20 mg), White LED (15 W), 100 °C, 45 min.

^a^Calculated by acid-base titration.

**Spectral information of EMF**

Calcd for C_8_H_10_O_3_: C, 62.33; H, 6.54; O, 31.13; found: C, 63.36; H, 6.51; O, 30.13.

**Fig. S1.** HPLC chromatogram of 5-HMF (standard), 5-EMF (standard), and synthesized products (EMF).

**Table S2.** Hypothetical high-loading tests (30 mg catalyst).

| Substrate (mmol HMF) | HMF (mg) | EMF Yield (%) | EMF Produced  (g) | g EMF / g Catalyst |
| --- | --- | --- | --- | --- |
| 1 | 126.11 | 0.95 | 25 | 5 |
| 5 | 630.55 | 1.41 | 78 | 21.1 |
| 10 | 1261.1 | 1.77 | 33 | 34.9 |

Reaction conditions: HMF (substrate), ethanol (4 mL), White LED (15 W), 100 °C, 75 min.

A


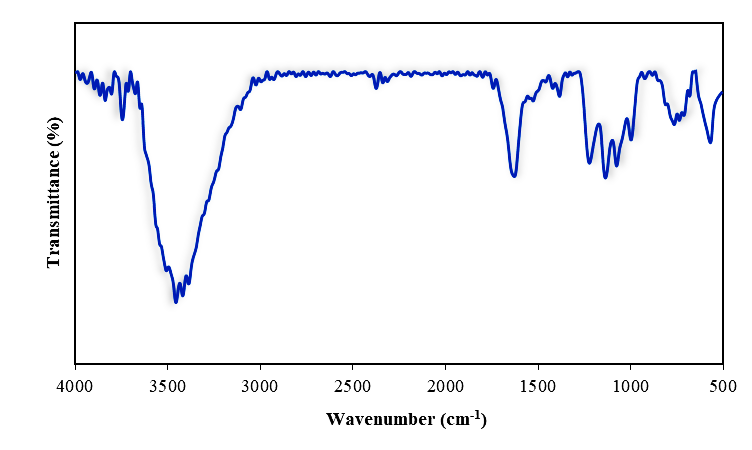


B

**Fig. S2.** XRD pattern (A) and FT-IR (B) of the spent Fe_3_O_4_@TiO_2_-diamine-SO_3_H catalyst after the reaction 5^th^ cycle.
